# A Tumor Progression Related 7-Gene Signature Indicates Prognosis and Tumor Immune Characteristics of Gastric Cancer

**DOI:** 10.3389/fonc.2021.690129

**Published:** 2021-06-14

**Authors:** Fen Liu, Zongcheng Yang, Lixin Zheng, Wei Shao, Xiujie Cui, Yue Wang, Jihui Jia, Yue Fu

**Affiliations:** ^1^ Department of Microbiology/Key Laboratory for Experimental Teratology of the Chinese Ministry of Education, School of Basic Medical Science, Cheeloo College of Medicine, Shandong University, Jinan, China; ^2^ Key Laboratory of Infection and Immunity of Shandong Province, School of Basic Medical Science, Cheeloo College of Medicine, Shandong University, Jinan, China; ^3^ Department of Implantology, School and Hospital of Stomatology, Cheeloo College of Medicine, Shandong University & Shandong Key Laboratory of Oral Tissue Regeneration & Shandong Engineering Laboratory for Dental Materials and Oral Tissue Regeneration, Jinan, China; ^4^ School of Basic Medical Science, Cheeloo College of Medicine, Shandong University, Jinan, China

**Keywords:** gastric cancer, tumor microenvironment, immunotherapy, WGCNA, prognosis

## Abstract

**Background:**

Gastric cancer is a common gastrointestinal malignancy. Since it is often diagnosed in the advanced stage, its mortality rate is high. Traditional therapies (such as continuous chemotherapy) are not satisfactory for advanced gastric cancer, but immunotherapy has shown great therapeutic potential. Gastric cancer has high molecular and phenotypic heterogeneity. New strategies for accurate prognostic evaluation and patient selection for immunotherapy are urgently needed.

**Methods:**

Weighted gene coexpression network analysis (WGCNA) was used to identify hub genes related to gastric cancer progression. Based on the hub genes, the samples were divided into two subtypes by consensus clustering analysis. After obtaining the differentially expressed genes between the subtypes, a gastric cancer risk model was constructed through univariate Cox regression, least absolute shrinkage and selection operator (LASSO) regression and multivariate Cox regression analysis. The differences in prognosis, clinical features, tumor microenvironment (TME) components and immune characteristics were compared between subtypes and risk groups, and the connectivity map (CMap) database was applied to identify potential treatments for high-risk patients.

**Results:**

WGCNA and screening revealed nine hub genes closely related to gastric cancer progression. Unsupervised clustering according to hub gene expression grouped gastric cancer patients into two subtypes related to disease progression, and these patients showed significant differences in prognoses, TME immune and stromal scores, and suppressive immune checkpoint expression. Based on the different expression patterns between the subtypes, we constructed a gastric cancer risk model and divided patients into a high-risk group and a low-risk group based on the risk score. High-risk patients had a poorer prognosis, higher TME immune/stromal scores, higher inhibitory immune checkpoint expression, and more immune characteristics suitable for immunotherapy. Multivariate Cox regression analysis including the age, stage and risk score indicated that the risk score can be used as an independent prognostic factor for gastric cancer. On the basis of the risk score, we constructed a nomogram that relatively accurately predicts gastric cancer patient prognoses and screened potential drugs for high-risk patients.

**Conclusions:**

Our results suggest that the 7-gene signature related to tumor progression could predict the clinical prognosis and tumor immune characteristics of gastric cancer.

## Introduction

Gastric cancer is the fifth most frequently diagnosed cancer and the third leading cause of cancer-related death worldwide (1). Many patients are diagnosed with advanced gastric cancer, and another 25–50% of patients will develop metastasis during the course of the disease (2). Despite continuous improvements in treatment, the 5-year survival rate of metastatic gastric cancer is only 5–20% (3–5). Immunotherapy has broad application prospects in gastric cancer, and immune checkpoint blockade is now established as a treatment for chemorefractory gastric cancer (6). The use of immunotherapy alone or in combination with other therapies can have a positive impact on the treatment of gastric cancer, but due to the high heterogeneity of gastric cancer, the response rate of patients during immunotherapy is not satisfactory (7, 8). Therefore, it is necessary to identify biomarkers and genetic characteristics to define the subgroup of gastric cancer patients most likely to respond to a specific immunotherapy (9, 10).

The tumor microenvironment (TME) is composed of cellular and noncellular components, including peripheral blood vessels, immune cells, fibroblasts, tumor stem cells, and extracellular matrix (ECM) (11). Studies have fully shown that tumor growth depends not only on the accumulation of abnormal genetic material in the original cancer cells but also on the TME, which provides conditions for the survival, growth and migration of cancer cells (12, 13). Immune cells in the TME, especially tumor infiltrating lymphocytes (TILs), have become prognostic and predictive factors for many solid tumors (14). In addition, immune cells in the TME are also important factors affecting the immunotherapy response (15), and TILs have been used as markers for the immunotherapy response. Immune cells in the TME play an important role in tumorigenesis, and tumor-related immune cells can antagonize or promote tumor progression, with the specific role depending on the composition and proportion of immune cells. Compared with traditional chemotherapy, immunotherapy mainly uses immune cells to specifically identify and attack cancer cells. Therefore, by analyzing the composition and proportion of immune cells in the tissues of gastric cancer patients, it is possible to evaluate whether the patient can benefit from immunotherapy.

At present, the American Joint Committee on Cancer (AJCC) stage is still the most basic prognostic prediction tool for gastric cancer, and a high stage indicates a poor prognosis. However, due to the high heterogeneity of gastric cancer, patients with the same tumor-node-metastasis (TNM) stage may have different prognoses (16). Similarly, differences in responses to immunotherapy among patients may also be related to their genetic and molecular backgrounds. Therefore, it is necessary to fully understand the specific characteristics of each patient, incorporate other important factors, and then conduct individualized treatment and prognosis prediction. Through the bioinformatics analysis of large-scale genomic or transcriptomic data, molecular markers related to the occurrence, development and prognosis of gastric cancer can be screened to provide reliable treatment targets for precision medicine, which has advantages in personalized treatment and prognosis prediction and broad prospects (17–22).

**Figure 1:**
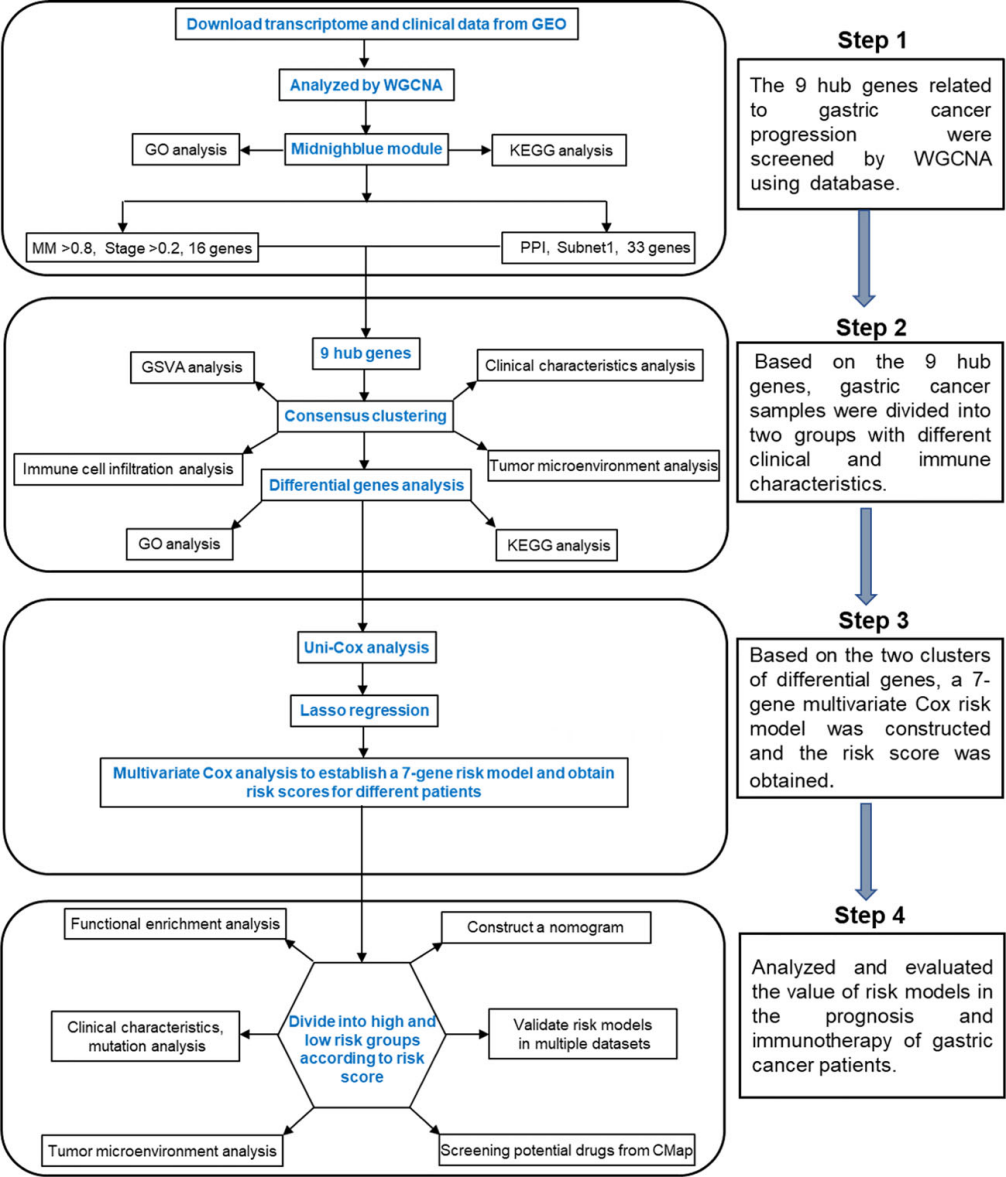
Detailed flow chart of this research.

In this study, we found a module related to tumor progression in the gastric cancer dataset by the weighted gene coexpression network analysis (WGCNA) method and identified nine hub genes. According to the hub genes, unsupervised clustering grouped the gastric cancer samples into two subtypes with different clinical and immune characteristics. We also explored the differences in gene expression patterns between the two subtypes. Finally, a 7-gene signature based on the differentially expressed genes was constructed. A nomogram based on the age, stage and risk score was established to provide theoretical guidance for clinical prognosis prediction. By analyzing the relationship between the risk score, TME and immune characteristics, it was found that high-risk patients were more suitable for immunotherapy, which provides theoretical support for the application of clinical immunotherapy. The flow chart of this research is shown in **Figure 1**.

## Materials and Methods

### Patients and Data Processing

Clinical samples for inclusion in this study were required to meet the following criteria: histologically confirmed as gastric adenocarcinoma, surgical resection of primary gastric cancer, age ≥18 years, with complete pathological, surgical, treatment, and follow-up data. Clinical samples collected in this study required a clinical diagnosis of gastric adenocarcinoma and no radiotherapy and chemotherapy prior to surgery. Detailed patient information in publicly available databases was available with reference to relevant research literature. According to our patient selection criteria, sixteen pairs of gastric cancer and adjacent normal tissues were collected from Shandong Cancer Hospital and frozen in liquid nitrogen until further analysis. Gastric cancer transcriptome data and clinical data GSE26901 (n = 109) (23), GSE15460 (n = 248) (3), GSE62254 (n = 300) (5), GSE15459 (n = 192), and GSE84437 (n = 433) were downloaded from the Gene Expression Omnibus (GEO) database (http://www.ncbi.nlm.nih.gov/geo/). The transcriptome data included the original data files (CEL files) and platform files. Gastric adenocarcinoma mutation data, survival data, and fragments per kilobase of transcript per million mapped reads (FPKM) transcriptome data were downloaded from The Cancer Genome Atlas (TCGA) (https://cancergenome.nih.gov/) and the cBioPortal database (http://www.cbioportal.org/), and there were 323 samples with transcriptome and survival data and 286 samples with transcriptome, survival and mutation data. The transcriptional-level differential expression analysis results of nine hub genes and seven genes used for modeling between normal tissues and tumor tissues were from GEPIA (http://gepia.cancer-pku.cn/index.html) (24). The survival curves of nine hub genes and seven genes used for modeling were from the Kaplan–Meier plotter (http://kmplot.com/analysis/index.php?p=background) (25).

The “affy” and the “impute” R packages in R/Bioconductor software were used for GEO data processing, and the “limma” package was used for differential gene expression analysis. We referred to Yang et al. (26) for the specific processing procedures. The ESTIMATE (Estimation of STromal and Immune cells in MAlignant Tumor tissues using Expression data) algorithm was applied to evaluate stromal and immune microenvironment infiltration (27), and the proportions of infiltrating stromal and immune cells in gastric cancer samples were quantified by stromal and immune scores using gene expression signatures. In this study, the immune score, stromal score, ESTIMATE score, and tumor purity were all obtained through the “estimate” R package in R software. The CIBERSORT algorithm was used to normalize the expression data to infer the absolute proportions of 22 kinds of infiltrating immune cells. CIBERSORT is a deconvolution algorithm that uses a set of reference gene expression values (547 genes) to predict the proportions of 22 immune cell types from a large number of tumor sample expression data by support vector regression (28). The infiltration levels of 22 immune cells were obtained through the CIBERSORT website (https://cibersort.stanford.edu/). The “maftools” R package was used to analyze the quantity and quality of gene mutations in the high-risk and low-risk groups, and the “GenVisR” package was used to draw a mutation waterfall chart. The Connectivity Map (CMap) database was used to find drugs that were negatively correlated with the input differential gene profile after acting on the cells (29) (https://portals.broadinstitute.org/cmap/).

### Construction of a Weighted Gene Coexpression Network

The GSE26901 dataset includes 109 gastric cancer patients and provides the patient sex, age, and tumor stage, which is suitable for the construction of a weighted gene coexpression network. The data matrix of gene expression in GSE26901 was constructed by using the **“**WGCNA**”** R package, and the top 25% of genes in tumor samples with the largest variance were selected as the input dataset for the subsequent WGCNA. To select the standard scale-free network, the sample hierarchical clustering method was used to detect and remove abnormal samples before selecting the appropriate soft threshold function. In the next stage, the adjacency matrix and topological overlap matrix (TOM) were constructed, the corresponding dissimilarity (1-TOM) was calculated, and dynamic tree cutting was used to complete the gene tree and module identification. The minimum module size was 30. Then, the module characteristic genes were fused by clustering, and the highly similar modules were merged. The degree of difference was less than 0.25, and the correlation between the module characteristic genes and the clinical phenotype of gastric cancer was calculated.

### Clustering and Enrichment Analysis

Consensus clustering is an algorithm that can be used to identify cluster members and their numbers in data sets (such as microarray gene expression). In this study, based on the expression values of the nine hub genes, we used the “ConsensusClusterPlus” software package to cluster samples into two clusters. Gene Ontology (GO) and Kyoto Encyclopedia of Genes and Genomes (KEGG) analyses were performed using the “ClusterProfiler” R package. The “GSVA” R software package was used for gene set variation analysis (GSVA) in different sample clusters (30). Gene set enrichment analysis (GSEA) was performed to investigate the functions correlated with different risk groups by GSEA 4.1.0, and the software was downloaded from the website for GSEA (http://www.gsea-msigdb.org/gsea/downloads). ssGSEA (single sample GSEA) analysis of gastric cancer samples based on 29 immune-related gene sets was performed using the “gsva” package, and scores of immune cell types, functions, and pathways were obtained for each sample. Hierarchical clustering of samples based on scores using the “sparcl” R package allowed the samples to be divided into high and low immune groups (30, 31).

### Core Network Identification

The genes in the midnight blue module were input into the Search Tool for the Retrieval of Interacting Genes/Proteins (STRING) website for protein-protein interaction (PPI) analysis, and PPI scores were obtained. Then, the results were imported into Cytoscape and analyzed using the MCODE plug-in. Finally, two core subnetworks (subnets) were obtained.

### Construction and Validation of the Risk Model

First, the GSE62254 and GSE15460 datasets were combined to eliminate batch effects using the “sva” R package. A total of 548 samples was obtained. In total, 276 samples were randomly selected as the training set, and the remaining 272 samples were used as the validation set. The “survival” R package was used to perform univariate Cox regression analysis of the differentially expressed genes between clusters 1 and 2, the “glmnet” R package was used for least absolute shrinkage and selection operator (LASSO) analysis, and the “survival” R package was used for multivariate Cox regression analysis to establish a risk model. The risk score was obtained using the “predict” function in R software, and the mathematical model of the risk score is as follows:

$Risk\;score\;=\;h_{0}{\left(t\right)}_{*}exp\left(\beta_{1}X_{1}+\;\beta_{2}X_{2}+\ldots +\beta_{n}X_{n}\right)$

where n is the representative number of modeling genes; β and X are the correlation coefficient and expression level of model gene prediction, respectively; and h_0_(t) is derived from the “predict” function.

### Construction and Assessment of the Nomogram

We used the “rms” R package to build the nomogram and the calibration chart. The calibration chart was used to evaluate the performance of the nomogram, and the “pROC” R package was used to draw the receiver operating characteristic (ROC) curve to evaluate the accuracy of the nomogram. Decision curve analysis (DCA) was employed to determine the clinical usefulness of the nomogram by quantifying the net benefits at different threshold probabilities using the “rmda” package in R software.

### RNA Extraction, QRT-PCR

Total RNA in clinical samples was extracted using the TRIzol method following the manufacturer’s protocol (Invitrogen, Carlsbad, CA, USA). Complementary DNA (cDNA) was synthesized using the PrimeScript™ RT Reagent Kit with gDNA Eraser (TaKaRa, Japan). The expression of the seven genes was verified by PCR using TB Green™ Premix Ex Taq™ (TaKaRa, Japan). The primers used in QRT-PCR assays are listed in **Supplementary Table 1**.

### Statistical Analysis

We used the “survival” R package to draw the survival curve in R software and perform statistical analysis. Time-dependent receiver operating characteristic (ROC) analysis and the calculation of the area under the curve (AUC) were performed by the “survcomp” and “survival” packages of R software. The comparison of integrated area under the curves (IAUC) used “iauc.comp” package. GraphPad Prism 8.0 was used to draw various bar graphs. The data were reported as the mean ± SEM. In this study, analyses between the two groups were performed using Student’s t-tests or Mann–Whitney tests. One-way analysis of variance was used to analyze the difference between multiple groups. Categorical variables in different groups were analyzed used the chi-square test by SPSS22.0. Spearman’s test was used to analyze the correlation between the two groups. Statistical significance was described as follows: n.s., not significant; *P < 0.05; **P < 0.01; and ***P < 0.001.

## Results

### WGCNA Identified the Modules Related to Tumor Progression and Further Screened the Hub Genes

**Figure 2:**
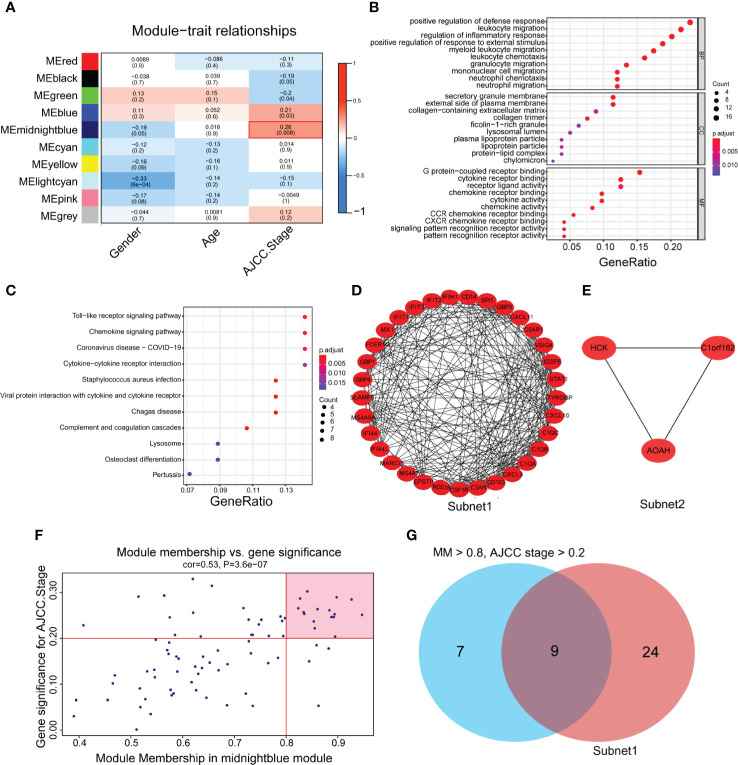
The identification of hub genes related to gastric cancer progression. **(A)** Heatmap of the correlations between module eigengenes and the clinical traits of gastric cancer. **(B)** Gene Ontology (GO) analysis of the genes in the midnight blue module (p < 0.05). **(C)** Kyoto Encyclopedia of Genes and Genomes (KEGG) analysis of the genes in the midnight blue module (P < 0.05). **(D, E)** Protein-protein interaction (PPI) core network of subnet 1 (MCODE score = 15.1) and subnet 2 (MCODE score = 3.0) in the midnight blue module. **(F)** Scatter plot genes in the midnight blue module. The vertical line represents the cutoff of module membership = 0.8, and the horizontal line represents the cutoff of gene significance for AJCC stage = 0.2. **(G)** The Venn diagram shows the intersection of the MCODE core network and the module membership (>0.8) and the significance of correlations with AJCC stage (>0.2) genes in the module.

To identify genes related to the progression of gastric cancer, a coexpression network was constructed by WGCNA in GSE26901. After removing five outlier samples, 104 samples were used to construct an adjacency matrix (**Supplementary Figure 1A**). In this study, we selected β = 9 as the soft thresholding power to achieve a scale-free network (**Supplementary Figures 1B, C** **)**. As a result, 10 gene coexpression modules were identified after using a merged dynamic tree cut (**Supplementary Figure 1D**). A network heatmap among 1,000 selected random genes was constructed to analyze the interaction relationships of the 10 modules (26), and it was clearly found that the genes within the module were highly correlated. In addition, the modules are also interrelated rather than independent of each other (**Supplementary Figure 1E**). By calculating the correlations between module eigengenes and clinical features, we found that the midnight blue module had the strongest correlation with the AJCC stage (**Figure 2A**). The heatmap shows the expression profiles of all genes in the midnight blue module (**Supplementary Figure 1F**).

To gain further insight into the function of genes in the midnight blue module, GO and KEGG analyses were performed. We detected enrichment in several biological process (BP) GO terms, such as positive regulation of defense response, leukocyte migration and regulation of the inflammatory response (**Figure 2B**). In terms of cellular components (CC), the secretory granule membrane, external side of the plasma membrane and collagen-containing extracellular matrix were enriched (**Figure 2B**). Moreover, some molecular function (MF) GO terms, such as G protein-coupled receptor binding, chemokine receptor binding and receptor ligand activity, were enriched (**Figure 2B**). Regarding KEGG pathway analysis, the Toll-like receptor signaling pathway, chemokine signaling pathway and cytokine–cytokine receptor interaction were mostly associated with these genes (**Figure 2C**).

To obtain the hub genes, we analyzed the PPI network of the genes in the midnight blue module, imported the results into Cytoscape software, processed them with the MCODE plug-in, and obtained two subnets, subnet1 and subnet2, under the condition of degree cutoff = 2 (**Figures 2D, E** **)**. We chose subnet 1 as the next research object (**Figure 2D**) because it had more genes (n = 33), complex networks and a high MCODE score. We considered the importance of genes in the midnight blue module and the relevance of clinical staging, and under the conditions of module membership >0.8 and significance of correlations with AJCC stage >0.2, 16 genes in the midnight blue module were selected (32) (**Figure 2F**). When the 16 genes of the midnight blue module and the above 33 genes of subnet 1 overlapped, a total of nine hub genes was obtained (**Figure 2G**).

To understand the role of the nine hub genes in the progression of gastric cancer, we analyzed the differential expression of these genes in cancer tissues and normal tissues in GEPIA. The results showed that *C1QA*, *CIQB*, *C1QC*, *CD14*, *FCER1G*, and *TYROBP* were highly expressed in gastric cancer tissues compared with normal tissues (**Supplementary Figure 2**), and there were no significant differences in *CD163*, *CSF1R*, and *MS4A6A*. Kaplan–Meier survival analysis in the Kaplan–Meier plotter showed that all hub genes except for *CD14* and *FCER1G* had obvious prognostic value (**Supplementary Figure 3**).

### Two Subtypes With Significant Differences in Terms of Clinical and Immune Characteristics and Biological Function Were Identified Based on the Consensus Clustering of Nine Hub Genes

**Figure 3:**
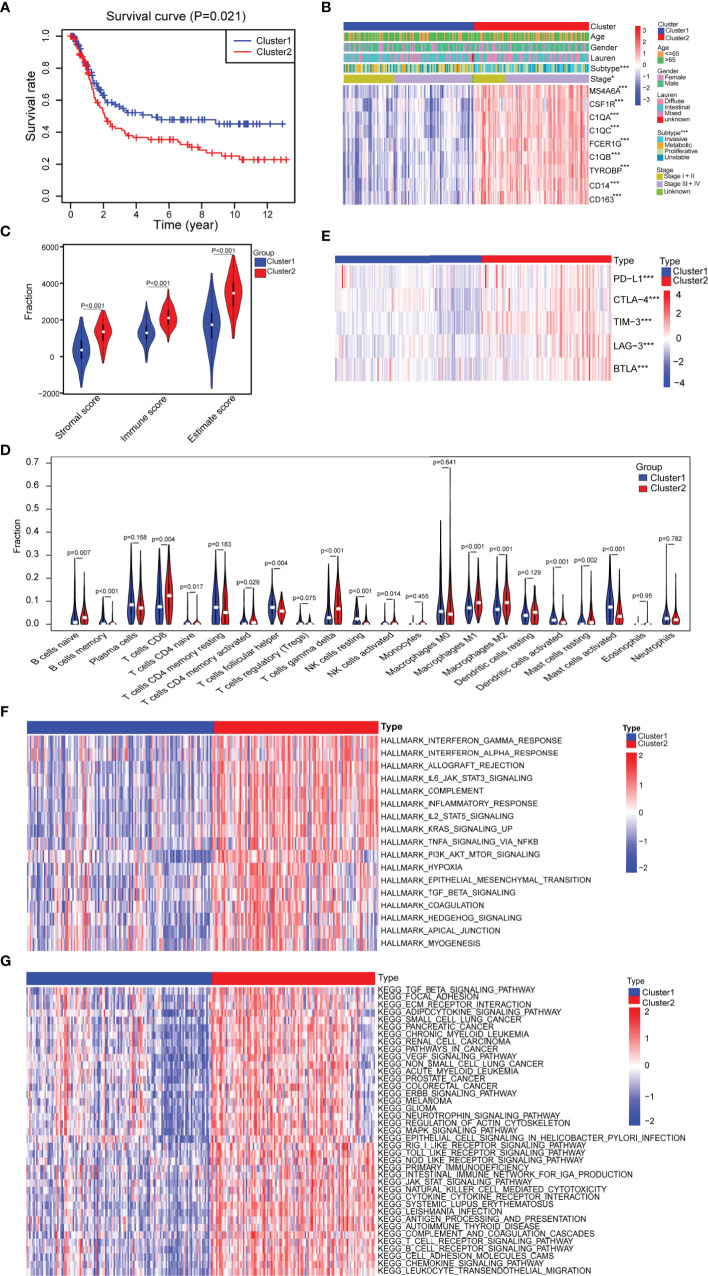
Assessment of the differences in clinical characteristics and immune components between the two subtypes. **(A)** Kaplan–Meier survival analysis of the two clusters (cluster 1 n = 132, cluster 2 n = 116; log-rank test). **(B)** Analysis of the differences in clinical characteristics between the two clusters (cluster 1 n = 132, cluster 2 n = 116; categorical variables, chi-square tests; continuous variable, Student’s t-tests). **(C)** Identification of the differences in the tumor microenvironment (TME) between the two clusters (cluster 1 n = 132, cluster 2 n = 116; Student’s t-tests). **(D)** Comparison of the difference in the number of immune cells between the two clusters (cluster 1 n = 132, cluster 2 n = 116; Wilcoxon tests). **(E)** Comparison of the expression levels of immune checkpoint molecules between the two clusters (cluster 1 n = 132, cluster 2 n = 116; Student’s t-tests). **(F)** Difference heatmap of GSVA-based HALLMARK enrichment analysis between the two clusters (false discovery rate (FDR) <0.05) (cluster 1 n = 132, cluster 2 n = 116; Student’s t-tests). **(G)** Difference heatmap of GSVA-based KEGG enrichment analysis between the two clusters (FDR <0.05) (cluster 1 n = 132, cluster 2 n = 116; Student’s t-tests). All data are from the GSE15460 dataset. Significant difference between the two groups: *P < 0.05; ***P < 0.001.

To further investigate whether the nine progression-related hub genes play a synergistic role in gastric cancer, we used the “ConsensusClusterPlus” software package to cluster patients according to the expression profiles of the nine genes in GSE15460. The patients were divided into two separate clusters (**Supplementary Figure 4A**). Next, we considered whether two was the best cluster number. The consensus cumulative distribution function (CDF) diagram shows that when the cluster number was 2, the CDF curve had the smallest slope (**Supplementary Figure 4B**). We used the “NbClust” software package to evaluate the optimal number of clusters and found that the optimal number of clusters was 2 (**Supplementary Figure 4C**). To further verify the classification, we evaluated the two clusters through principal component analysis (PCA), and the results showed that the two clusters could still be separated (**Supplementary Figure 4D**). To assess the clinical significance of the classification, we compared the differences in prognosis and clinical characteristics between the two subtypes of gastric cancer patients. We found that compared with cluster 1 patients, cluster 2 patients had significantly worse prognoses (**Figure 3A**). The distribution of classifications reported before of the two clusters of patients was significantly different. Metabolic and proliferative types were mainly observed in cluster 1, while the invasive subtype was observed more frequently in cluster 2 (**Figure 3B**) (33). Regarding the stage, there were higher proportions of stages I and II patients in cluster 1 and higher proportions of stages III and IV patients in cluster 2 (**Figure 3B**). We then analyzed the expression of several classic invasion and migration markers in the two clusters of patients and found that *VIM*, *SNAI1*, *SNAI2*, *TWIST1*, *MMP2*, *MMP7*, and *MMP9* were highly expressed in cluster 2 patients (**Supplementary Figure 4E**). The heatmap results showed that compared with that in cluster 1, the expression of the nine hub genes in cluster 2 was significantly higher (**Figure 3B**). Since the TME plays an important role in cancer progression, we first used the “estimate” R package in the GSE15460 dataset to obtain the TME scores of the two sample clusters and then compared them. The immune score, stromal score, and estimated score values showed that there were more infiltrating immune cells and stromal cells in cluster 2 than in cluster 1 (**Figure 3C**). Conversely, cluster 1 had a higher tumor purity than cluster 2 (**Supplementary Figure 4F**). In view of the difference in the level of immune cell infiltration between the two types of patients, we also compared the levels of 22 kinds of immune cells between the two groups. We found that there were many kinds of immune cells with significantly different infiltration levels in the two groups. For example, cluster 2 exhibited greater infiltration of CD8+ T cells, CD4+ memory-activated cells, M1 macrophages and M2 macrophages, and cluster 1 exhibited greater infiltration of B cells and follicular helper T cells (**Figure 3D**). In addition, we found that there were differences in the expression of multiple immunosuppressive checkpoint molecules between the two groups, and their expression levels in cluster 2 were significantly higher than those in cluster 1 (**Figure 3E**). To compare the biological function differences between the two clusters, we conducted GSVA analysis. The results of hallmark and KEGG enrichment analysis showed that a variety of signaling pathways related to the immune response, signal transduction, epithelial-mesenchymal transition (EMT), hypoxia and tumor progression pathways were enriched in cluster 2 samples (**Figures 3F, G** **)**.

In short, based on nine hub genes, the samples can be divided into two subtypes by consensus clustering, and the subtypes have obvious differences in clinical and immune characteristics and biological functions.

### A Risk Model With Prognostic Value Was Constructed Based on Seven Differentially Expressed Genes Between the Two Clusters

To explore the hidden mechanism that drives the clinical and immune characteristics and biological function differences between the two clusters, we analyzed the differences between the mRNA expression profiles of the two clusters of samples in GSE15460, and in total, 200 differentially expressed genes were obtained (false discovery rate (FDR) <0.05, |log2FoldChange| >1), of which 174 were upregulated and 26 were downregulated in cluster 2 *versus* cluster 1 (**Supplementary Figure 5A** and **Supplementary Table 2**). The heatmap shows the top 20 differentially expressed genes that were upregulated and downregulated in cluster 2 (**Supplementary Figure 5B**). Subsequently, GO and KEGG enrichment analyses were performed on the differentially expressed genes. In the GO and KEGG enrichment analysis, the response to chemokines, extracellular matrix, CXCR chemokine, chemokine signaling pathway and other signaling pathways related to immune cell response and migration were enriched (**Supplementary Figures 5C–F**). Based on this finding, we speculated that these differentially expressed genes may play a role in immune cell migration and the immune response.

**Figure 4:**
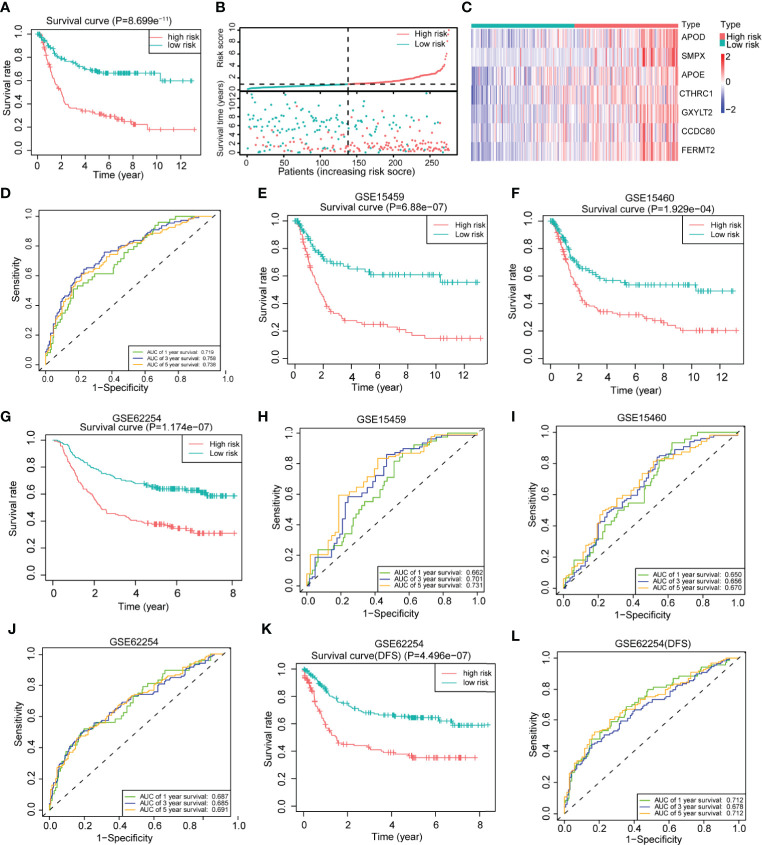
Construction of a 7-gene signature for gastric cancer based on differentially expressed genes between the two clusters. **(A)** Kaplan–Meier survival analysis of the different patient risk groups (high-risk n = 138, low-risk n = 138; log-rank test). **(B)** The survival time, survival status and risk score of patients in different risk groups (high-risk n = 138, low-risk n = 138). **(C)** Heatmaps of the expression of the seven model genes in different risk groups of patients (high-risk n = 138, low-risk n = 138). **(D)** Time-dependent receiver operating characteristic (ROC) analysis of the risk score in gastric cancer patients. **(E)** An overall survival curve was drawn for the GSE15459 datasets based on the same cutoff value used to obtain the training set risk score (high-risk n = 100, low-risk n = 92). **(F)** An overall survival curve was drawn in GSE15460 datasets based on the same cutoff value used to obtain the training set risk score (high-risk n = 127, low-risk n = 121). **(G)** An overall survival curve was drawn for the GSE62254 datasets based on the same cutoff value used to obtain the training set risk score (high-risk n = 138, low-risk n = 162). **(H–J)** Plot of time-dependent survival ROC curves in different datasets. **(K)** A disease-free survival (DFS) curve was drawn for the GSE62254 dataset according to the same cutoff value used to obtain the training set risk score (high-risk n = 138, low-risk n = 162). **(L)** Plot of the time-dependent ROC curve for DFS. The data for **(A–D)** are from the training set. Data for **(E, H)** are from the GSE15459 dataset; **(F, I)** are from GSE1540; and **(G, J–L)** are from the GSE62254 dataset.

To further explore the clinical value of the differentially expressed genes, we first performed univariate Cox regression analysis on the above 200 differentially expressed genes in the training set and obtained 88 genes with prognostic value (P <0.01) (**Supplementary Table 3**). The LASSO regression analysis method was used to remove the strong collinearity genes (**Supplementary Figures 6A, B** **)**, and finally, 10 genes were obtained in the training set (**Supplementary Table 4**). After multivariate Cox regression, seven genes were obtained by further optimization analysis: *APOD*, *APOE*, *CCDC80*, *CTHRC1*, *FERMT2*, *GXYLT2*, and *SMPX* (all seven genes mentioned below refer to these seven genes). Furthermore, we used the “predict” function of R to construct a 7-gene signature to estimate the risk score of each patient based on the mRNA expression level of each gene weighted by the multivariate Cox regression coefficient (**Supplementary Table 5**). Based on the median risk score, patients in the training set were divided into high- and low-risk groups. Kaplan–Meier survival analysis showed that patients in the high-risk group had worse prognoses than those in the low-risk group (**Figure 4A**). The heatmap of the survival time, survival status, and risk score showed the distribution of patients into different risk groups (**Figure 4B**). The expression heatmap showed that the seven modeled genes were highly expressed in the high-risk group (**Figure 4C**). The time-dependent ROC curve analysis based on the risk score showed that the 1-year, 3-year and 5-year area under the curve (AUC) values were 0.719, 0.758, and 0.738 (**Figure 4D**), respectively, indicating that the risk score can predict survival with relatively high accuracy. We next verified the risk model in the verification set. Kaplan–Meier survival analysis showed that there were also significant differences in the survival of patients in the high- and low-risk groups in the validation set, and the high-risk group had a worse prognosis (**Supplementary Figure 6C**). The distribution heatmap of the risk score, survival time, and survival status shows the distribution of patients into different risk groups in the validation set (**Supplementary Figure 6D**). The expression heatmap of the validation set also showed that the expression trends of the seven genes used for modeling were consistent with those of the training set (**Supplementary Figure 6E**). The time-dependent ROC curve of the risk validation set is shown (**Supplementary Figure 6F**). In addition, to further verify the universality of the risk model, we verified it using several gastric cancer datasets. For the GSE15459, GSE15460, GSE62254, GSE84437 and TCGA datasets, the Kaplan–Meier overall survival curve showed poor prognoses in the high-risk group (**Figures 4E–G** and **Supplementary Figures 6G, H** **)**, and the time-dependent ROC curve showed the accuracy of the survival curve for predicting survival at different times (**Figures 4H–J** and **Supplementary Figures 6I, J** **)**. The disease-free survival (DFS) curve yielded the same results as the overall survival curve (**Figure 4K**), and the ROC curve showed the accuracy of the model in predicting the DFS rate at different times in the GSE62254 dataset (**Figure 4L**).

**Figure 5:**
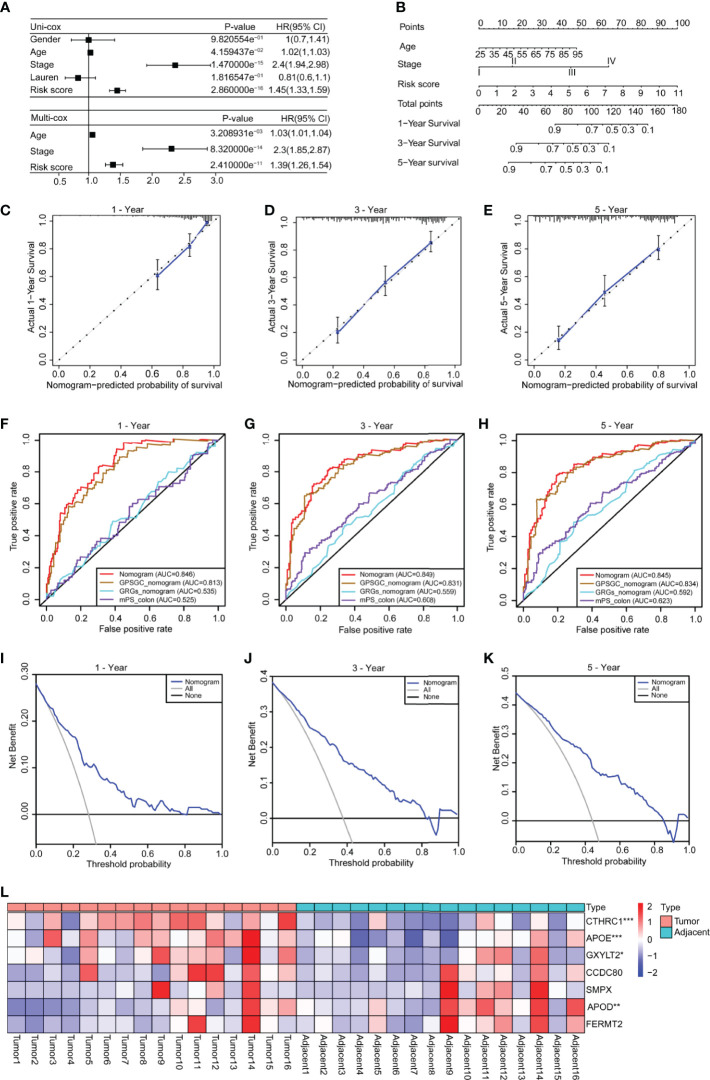
Construction of a nomogram based on the age, stage and risk score. **(A)** Univariate Cox analysis was used to analyze the clinical factors and risk score, and multivariate analysis was used to analyze the significant factors from the univariate Cox analysis. **(B)** A nomogram for clinical diagnosis was constructed based on clinical characteristics and the risk score. **(C–E)** The calibration plots for predicting recurrence at 1, 3, and 5 years. The X-axis represents the predicted recurrence probability from the nomogram, and the y-axis represents the actual recurrence probability. **(F–H)** Time-dependent ROC analysis of gastric cancer patient survival was used to evaluate the predictive accuracy of our nomogram and compare it with other previously developed and validated models. The area under the curve (AUC) was calculated and compared (Mann–Whitney tests). **(I–K)** Decision curve analysis of the nomogram for 1-, 3- and 5-year risk. The x‐axis represents the threshold probability, and the y‐axis represents the net benefit. The black line represents the assumption that no patients died at 1, 3, or 5 years. The gray line represents the assumption that all patients die at 1, 3, or 5 years. The blue dotted line represents the prediction model of the nomogram. All data are from the training set. **(L)** The heatmap shows the results of real-time fluorescent quantitative PCR for detecting the mRNA levels of the seven genes in 16 pairs of gastric cancer and adjacent normal tissues (n = 16, paired Student’s t-tests). Significant difference between the two groups: *P < 0.05; **P < 0.01; ***P < 0.001.

To explore the clinical value of the risk score, we combined clinical indicators, including the sex, age, stage, and Lauren classification, to perform univariate Cox regression analysis in the training set and found that the age, stage, and risk score had significant prognostic significance (**Figure 5A** **)**. We also conducted univariate Cox analysis on different validation sets and found that the risk score had prognostic significance in these datasets (**Supplementary Figures 7A–G**). The results of multivariate Cox regression analysis showed that the risk score can be used as an independent prognostic factor (**Figure 5A**).

To facilitate clinical application, we constructed a nomogram in the training set, according to the results of univariate Cox regression analysis in training and validation sets, which integrates the age, stage, and risk score (**Figure 5B**). The line segments in the three calibration graphs are all close to the 45° line, indicating that the nomogram shows good a prediction performance at 1, 3, and 5 years (**Figures 5C–E**). In addition, calculations revealed that the nomogram concordance index was 0.766, and the 95% confidence interval (CI) was 0.730–0.801. ROC analysis was used to evaluate the predictive accuracy of the nomogram, and the area under the curve (AUC) values of the 1-, 3-, and 5-year line graphs were 0.846, 0.849, and 0.845, respectively (**Figures 5F–H**). The decision curve showed that at 1, 3, and 5 years, the threshold probability was 3–79%, 4–83%, and 5–85%, respectively, and within this range, and the nomogram was used to predict survival more accurately (**Figures 5I–K**). We also compared our nomogram with three previously developed and validated prognostic models of gastric cancer, namely, GPSGC nomogram (34), GRGs nomogram (35), and mPS_colon (36). The time-dependent ROC curve analysis of our nomogram, GPSGC nomogram, GRGs nomogram, mPS_colon showed that the 1-, 3-, and 5-year areas under the curve (AUC) values were 0.846, 0.813, 0.535, and 0.525; 0.849, 0.831, 0.559, and 0.608; 0.845, 0.834, 0.592, and 0.623 (**Figures 5F–H**). Compared with GPSGC nomogram (Mann–Whitney tests; P <0.001), GRGs nomogram (Mann–Whitney tests; P <0.001) and mPS_colon (Mann–Whitney tests; P <0.001), our nomogram has a larger area under the curve (**Figures 5F–H**). In short, these results show that our nomogram has a good predictive performance and clinical application value.

To understand the roles of the seven genes in the progression of gastric cancer, we detected the mRNA expression levels of seven genes in 16 pairs of gastric cancer and adjacent normal tissues. The results showed that *APOE*, *CTHRC1*, and *GXYLT2* were highly expressed in gastric cancer tissues compared with adjacent normal tissues (**Figure 5L** and **Supplementary Figures 8B, D, F** **)**, while *APOD* was expressed at low levels in gastric cancer tissues (**Figure 5L** and **Supplementary Figure 8A**), and there were no significant differences in *CCDC80*, *FERMT2*, and *SMPX* (**Supplementary Figures 8C, E, G** **)**. We also analyzed the expression differences of these seven genes in cancer tissues and normal tissues in GEPIA, which were consistent with our detection results (**Supplementary Figures 8H–N** **)**. Kaplan–Meier survival analysis in the Kaplan–Meier plotter showed that all genes had obvious prognostic value and that high expression indicated a poor prognosis (**Supplementary Figure 9**). The circle graph shows the chromosomal location of the seven genes involved in the model (**Supplementary Figure 10A**). The bar chart shows the genetic alteration rate of the seven signature genes and their distribution in TCGA patients (**Supplementary Figure 10B**), and the results show that the genetic alteration rates of *APOD*, *CCDC80*, and *CTHRC1* were greater than 5% (**Supplementary Figure 10B**).

Overall, a gene signature was constructed based on seven differentially expressed genes that clustered patients into high- and low-risk groups with different prognoses, and a nomogram was constructed based on the age, stage, and risk score and had a good ability to predict prognoses.

### There Were Significant Differences in Clinical Characteristics and Biological Functions Between Different Risk Groups

**Figure 6:**
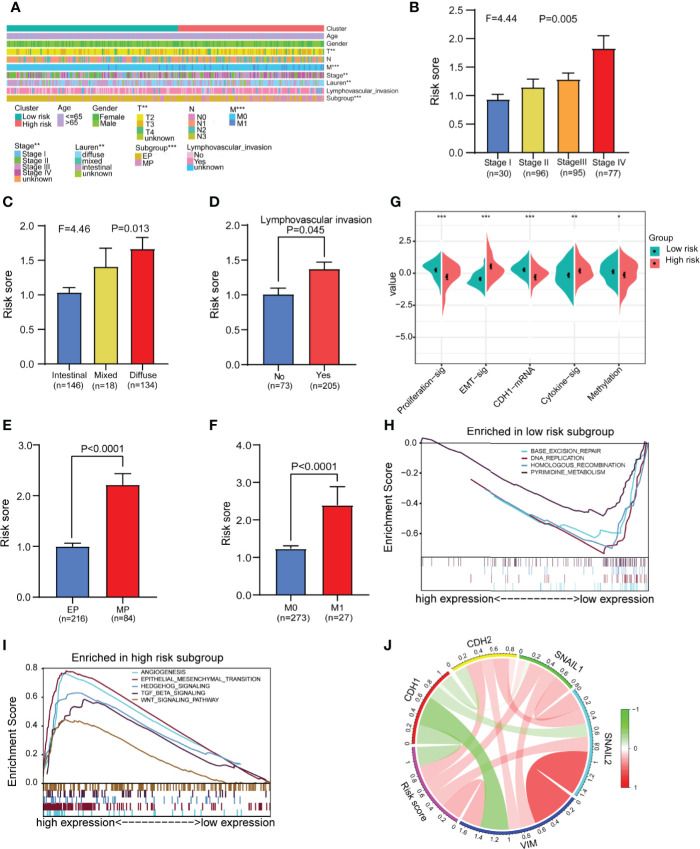
Analysis of differences in clinical characteristics and functional enrichment of patients in different risk groups. **(A)** The distributions of clinicopathological features were compared between the low-risk and high-risk groups (high-risk n = 138, low-risk n = 162 chi-square test). **(B, C)** Comparison of the risk scores of patients with different clinical stages and Lauren types (one-way analysis of variance). **(D)** Comparison of risk scores between patients with negative and positive lymphatic invasion status (Student’s t-test). **(E)** Comparison of the risk score of patients with epithelial phenotype (EP) and mesenchymal phenotype (MP) subtypes (Student’s t-test). **(F)** Comparison of the risk score of patients with no metastasis (M0) and metastasis (M1) (Student’s t-test). **(G)** Comparison of the different molecular signatures in the high- and low-risk groups (high-risk n = 138, low-risk n = 162 Student’s t-tests). **(H)** Gene set enrichment analysis (GSEA) in the low-risk group (n = 162, permutation tests P < 0.05, FDR < 0.25). **(I)** Gene set enrichment analysis (GSEA) in the high-risk group (n = 138, permutation tests P < 0.05, FDR < 0.25). **(J)** Analysis of the correlation between the risk score and EMT marker expression in gastric cancer patients (Pearson correlation coefficient). Data for **(A–J)** are from the GSE62254 dataset. Significant difference between the two groups: *P < 0.05; **P < 0.01; ***P < 0.001.

We analyzed and compared the clinical characteristics of patients in different risk groups in the GSE62254 dataset. The heatmap shows that the distributions of the T stage, M stage, tumor stage, Lauren classification, lymphovascular invasion and subgroup in the high- and low-risk groups were significantly different (**Figure 6A**). We found that the risk score was significantly altered among samples of different stages (**Figure 6B**), with a higher stage indicating a higher risk score. Compared with other subtypes, diffuse-type disease was related to a higher risk score (**Figure 6C** **)**. Patients with lymphatic invasion had a higher risk score than patients without lymphatic invasion (**Figure 6D**). Mesenchymal phenotype (MP) patients had a higher risk score than epithelial phenotype (EP) patients (**Figure 6E**). Metastasis (M1) patients had a higher risk score than no metastasis (M1) patients (**Figure 6F**). We also compared several common molecular phenotypic signatures, and the results showed that proliferation, cadherin-1 (*CDH1*) expression, and methylation signatures were strong indicators of a low risk, while EMT and cytokine signatures were strong indicators of a high risk (**Figure 6G**). Based on this finding, we speculate that most patients in the low-risk group have early-stage cancer, in which cell proliferation is a dominant feature, while most patients in the high-risk group have advanced-stage disease, which has invasion and migration characteristics. To verify our hypothesis and explore the differences in biological function between different risk groups in driving the progression of gastric cancer, we conducted GSEA in GSE62254. The results showed that the gene set enriched in low-risk samples was related to DNA replication and repair pathways (**Figure 6H**), while the gene set enriched in high-risk samples was related to cancer and tumor metastasis-related pathways (**Figure 6I**).

To further confirm the relationship between the risk score and EMT, we assessed the correlations between EMT markers and the risk score in GSE62254. The circle graph results show that the risk score is positively correlated with the expression of EMT-promoting molecules *CDH2*, *SNAIL1*, *SNAIL2*, and *VIM* and negatively correlated with the expression of the EMT-inhibiting molecule *CDH1* (**Figure 6J**). We also compared gene mutations in different risk groups in the TCGA dataset but found no difference in the total mutation load (**Supplementary Figures 11A–C**).

### The Immune Characteristics of Patients in the High- and Low-Risk Groups Were Significantly Different

**Figure 7:**
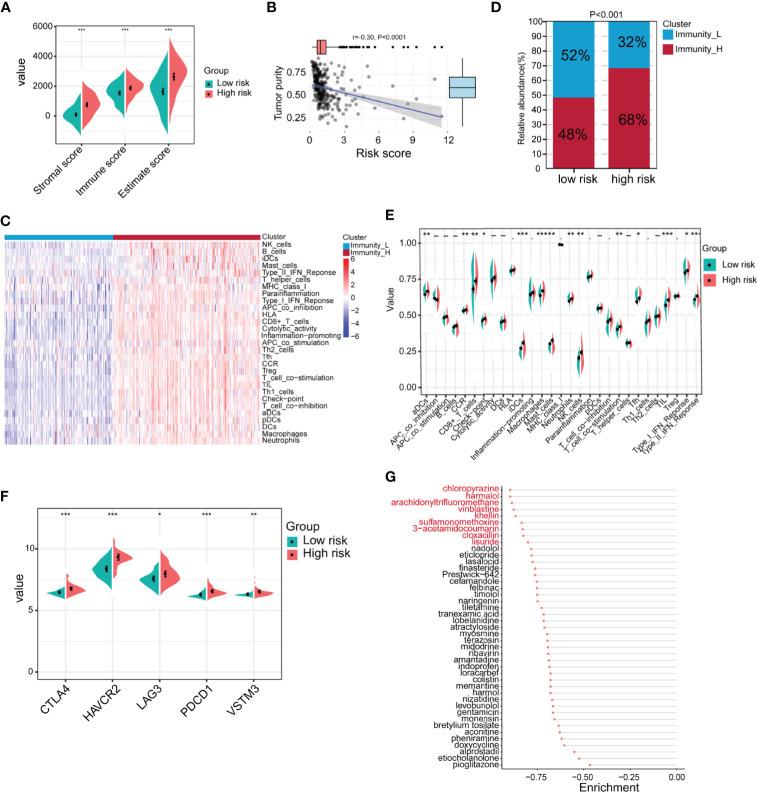
Identification of the immune characteristics of the high- and low-risk groups. **(A)** Differences in the TME between the high- and low-risk groups were identified (high-risk n = 138, low-risk n = 162; Student’s t-tests). **(B)** Correlation analysis of tumor purity and risk scores (Pearson correlation coefficient). **(C)** Chi-square analysis of high- and low-risk groups and different immune clusters (Immunity_L n = 128, Immunity_H n = 172 Chi-square test). **(D)** The gastric cancer samples were divided into two immune clusters by ssGSEA based on 29 immune signatures (Immunity_L n = 128, Immunity_H n = 172; chi-square test). **(E)** Analysis of differences in the expression of 29 immune signatures in the high- and low-risk groups (Student’s t-tests). **(F)** Expression analysis of immune checkpoints in different risk groups (high-risk n = 138, low-risk n = 162; Student’s t-tests). **(G)** The Connectivity Map (CMap) database was used to screen potential drugs for the treatment of high-risk patients. Drugs marked in red represent enrichment ≤0.8. Data for **(A–G)** are from the GSE62254 dataset. Significant difference between the two groups: *P < 0.05; **P < 0.01; ***P < 0.001.

Because the TME is closely related to EMT, we next compared the differences in the TME between the high-risk and low-risk groups in GSE62254. The results showed that the immune score, stromal score and ESTIMATE score of the high-risk group were significantly higher than those of the low-risk group (**Figure 7A**). Tumor purity was significantly negatively correlated with the risk score (**Figure 7B**). Next, we used ssGSEA to divide the samples into high-immune score and low-immune score groups based on 29 immune signatures in GSE62254 (**Figure 7C**). The results of the chi-square test showed that most patients in the high-risk group had high immune scores, and there were significant differences between the high-immune score group and the low-immune score group (**Figure 7D**). We further compared the differences in 29 immune signatures in the high- and low-risk groups and found that most of the signatures, such as immune response-related signatures, CD8+ T cells, NK cells, checkpoints, TILs, and the IFN response, were expressed at higher levels in the high-risk group (**Figure 7E**). We further compared the expression levels of immune checkpoint molecules in the high- and low-risk groups. The expression level of immune checkpoint molecules in the high-risk group was significantly higher than that in the low-risk group (**Figure 7F**). The above results suggest that patients in the high-risk group are more likely to benefit from immunotherapy than those in the low-risk group.

In addition to immunotherapy, we further explored other potential drugs that could be used for the treatment of patients in the high-risk group. We compared the transcriptome data of samples from the high- and low-risk groups. Under the conditions of |log2FoldChange| >0.585 (FoldChange >1.5) and FDR <0.05, in total, 440 genes were upregulated and 49 genes were downregulated in the high-risk group in GSE62254 (**Supplementary Table 6**). We imported the differentially expressed genes into the CMap database and screened 43 potential drugs that could be used to treat high-risk patients (**Supplementary Table 7** and **Figure 7G**). Among the drugs with enrichment ≤0.8 were chloropyrazine, harmalol, arachidonyltrifluoromethane, vinblastine, khellin, sulfamonomethoxine, 3-acetamidocoumarin, cloxacillin, and lisuride (**Figure 7G**). Vinblastine is a clinically used antitumor drug, harmalol has an antitumor effect *in vitro* (37), and khellin analogs can serve as new potential pharmacophores for epidermal growth factor receptor (EGFR) inhibitors (38), indicating that these drugs may be beneficial for the treatment of high-risk patients. The antitumor activity of the other drugs needs to be further studied.

## Discussion

Many patients have advanced gastric cancer at the time of diagnosis and miss the optimal treatment period; thus, their prognosis is relatively poor. At present, the AJCC stage is still the most common method of determining the prognosis of patients with gastric cancer. However, patients may still have different survivals with the same TNM stage, which may be due to different molecular characteristics of the tumors. Therefore, it is very important to develop a more sensitive prognostic diagnostic method according to the molecular characteristics of gastric cancer patients to identify new prognostic markers. The purpose of this study was to identify molecular signatures that can help predict prognoses and evaluate potential immunotherapy benefits. In this study, we used the mRNA expression and clinical data of gastric cancer samples in a public dataset for WGCNA and obtained a midnight blue module that was positively correlated with tumor progression. After further optimization and screening, we found the following nine hub genes, which are listed in order of impact: *C1QA*, *C1QB*, *C1QC*, *CSF1R* (colony-stimulating factor 1 receptor), *FCER1G* (Fc fragment of IgE receptor Ig), *CD14*, *MS4A6A* (membrane-spanning 4-domain subfamily A member 6A), scavenger receptor cysteine-rich type 1 protein M130 (*CD163*), and *TYROBP* (*TYRO* protein tyrosine kinase-binding protein). Among them, *C1QA*, *CIQB* and *C1QC* together form C1q to perform biological functions (39). C1q is an activator of the classical complement pathway, but there are studies showing that C1q can promote tumor proliferation and migration by interacting with the TME (40, 41), and this effect does not depend on the complement pathway. As a receptor on the cell membrane surface, *CSF1R* activates different signaling pathways by binding to different ligands to play a role in a variety of physiological and pathological processes, including tumorigenesis (42, 43). Studies have shown that *FCER1G* participates in a variety of immune functions and can be used as a prognostic marker for a variety of cancers (44–46). As a key component of the Toll-like receptor (TLR) signaling pathway, *CD14* can promote tumor occurrence and development by regulating the activation of different signaling pathways in tumor cells or tumor infiltrating immune cells (47–50). Research on *MS4A6A* is mainly focused on Alzheimer’s disease, and there are also current studies showing that it can be used as a prognostic marker for tumors (51, 52). *CD163* is a type I membrane protein and a member of the scavenger receptor superfamily. It is the most specific monocyte and macrophage marker currently in use, and it mainly plays a role in inflammation. Recent studies have shown that high expression of *CD163* is related to a poor prognosis in breast cancer (53) and glioma (54) patients. *TYROBP* is mainly involved in immune signaling pathways, but an increasing number of studies have shown that *TYROBP* can be used as a prognostic marker for cancer (55, 56). We found that the hub nine genes were mainly located in the extracellular matrix and cell membrane, which may indicate that they promote tumor progression by regulating the tumor microenvironment.

We divided the samples into two clusters based on the nine hub genes and found that compared with cluster 1 patients, cluster 2 patients had a worse prognosis. We compared their clinical characteristics and found that the proportions of patients with invasive subtypes and high tumor grades were higher in cluster 2, which explains why cluster 2 patients have a poor prognosis. We also found that the expression of invasion and migration markers in cluster 2 was increased significantly compared with that in cluster 1. Studies have shown that the TME can cause tumor cells to undergo EMT, thereby promoting tumor cell invasion and migration. However, the TME results showed that cluster 2 patients had higher TME component levels and lower tumor purity. This suggests that cluster 2 has a better prognosis, but we speculate that this may be because in cluster 2 patients, most immune cells are shielded from the outside of the solid tumor and cannot exert an immune killing effect due to the EMT of tumor cells. Similar results were found in previous studies on gastric cancer and other cancers (57–60). Studies have shown that the activation of the matrix in the TME can inhibit T cell activity (61). Studies have also shown that tumor-associated macrophages (TAMs) are negatively related to tumor prognoses. TAMs activate the tumor EMT process through the TGF-β signaling pathway and can also maintain the mesenchymal characteristics of tumor cells (62). TAMs have been shown to be similar to M2 macrophages (63). The analysis of the infiltration levels of 22 immune cells in this study showed that the infiltration of M2 macrophages was significantly higher in cluster 2 patients than in cluster 1 patients, which suggests that M2 macrophages could facilitate EMT and indicate a poor prognosis in cluster 2 patients. On the other hand, although cluster 2 exhibited high infiltration levels of immune cells that are beneficial to the immune response, such as CD8+ T cells, CD4+ T cells, and NK cells, the expression level of the immune suppression checkpoint in cluster 2 was higher, which led to immunosuppression. The GSVA results showed that cluster 2 samples exhibited activation of TGF-β signaling, EMT, hypoxia, and various cancer pathways. These results indicate that under the influence of the internal and external environment, the signaling pathway of tumor progression is activated, which leads to invasion and metastasis in cluster 2 patients, resulting in a poor prognosis.

In this study, we constructed a 7-gene signature. The seven genes were apolipoprotein D (*APOD*), apolipoprotein E (*APOE*), coiled-coil domain-containing protein 80 (*CCDC80*), collagen triple helix repeat-containing protein 1 (*CTHRC1*), fermitin family homolog 2 (*FERMT2*), glucoside xylosyltransferase 2 (*GXYLT2*), and small muscular protein (*SMPX*). *APOD* is an apolipoprotein, and recent studies have shown that it can be used as a prognostic marker for breast cancer (64, 65). Studies have shown that *APOE* can not only promote the migration of gastric cancer cells by activating the PI3K-Akt signaling pathway but can also serve as a diagnostic marker for gastric cancer (66, 67). *CCDC80* can mediate the regulation by focal adhesion kinase (FAK) of the migration of melanoma cells and can also exert a tumor suppressor effect in thyroid cancer (68, 69). Previous studies have shown that *CTHRC1* can promote the metastasis of colorectal cancer, ovarian cancer, gastric cancer, and cervical cancer (70–73). *FERMT2* is a scaffolding protein that has been reported to promote the proliferation and migration of esophageal squamous cell carcinoma (74). *GXYLT2* promotes the proliferation and migration of human cancer cells by regulating the *NOTCH* signaling pathway (75). *SMPX* is a small muscle protein located in the nucleus. It has been reported to be involved in the formation of hearing (76), but it has not been reported in tumor studies. In this study, we also found that the high expression of these seven genes is related to a poor prognosis in gastric cancer patients, and based on this and previous reports, we speculate that these seven genes can promote the progression of gastric cancer.

According to the risk score obtained from the above risk model, gastric cancer patients were divided into high- and low-risk groups. The prognosis of patients in the high-risk group was worse. A comparison of clinical features revealed that patients in the high-risk group had a higher stage, more lymphatic invasion, and a higher frequency of diffuse-type disease. This shows that patients in the high-risk group have the characteristics of invasion and metastasis, which leads to a poor prognosis. Further research revealed that patients in the high-risk group had EMT characteristics, while patients in the low-risk group had proliferative characteristics. GSEA also showed that EMT, angiogenesis, and multiple tumor progression pathways were activated in the high-risk group. In the low-risk group, signaling pathways related to proliferation, such as base excision repair, DNA replication, homologous recombination and pyrimidine metabolism, were activated. This suggests that conventional chemotherapy drugs that induce DNA damage and cell cycle arrest may be more suitable for patients in the low-risk group. TME analysis showed that the high-risk group had a higher level of immune cell infiltration. Further analysis revealed that the high-risk group had higher levels of CD8+ T cells, TILs, NK cells, B cells and other immune activation-related cells and had higher immune suppression checkpoint expression levels, which means that the high-risk group may benefit from immunotherapy. However, studies have shown that activation of the EMT, TGF-β and angiogenesis pathways can inhibit T cell activity (57). This can also explain why patients in the high-risk group have a poor prognosis despite abundant immune cell infiltration. Therefore, the combination of EMT, TGF-β and angiogenesis pathway inhibitors during immunotherapy may yield greater benefits in patients. Based on CMap data, we also found several drugs with the potential to treat high-risk patients, but the exact efficacy of the drugs needs to be further confirmed by *in vivo* and *in vitro* trials.

In this study, we obtained a 7-gene signature by analyzing transcriptome data and clinical data, and we validated the accuracy of the risk model in multiple datasets. A large number of clinical samples and prospective studies are still needed to evaluate the value of the risk model in predicting the prognosis and immune response of gastric cancer patients and to determine the optimal cutoff value.

Above all, we developed a 7-gene signature related to tumor progression in our research. The nomogram constructed from the prognostic model risk score combined with the age and stage can predict prognoses well. In addition, according to the risk model, patients with gastric cancer can be divided into two groups with different clinical and immune characteristics, and the high-risk group is more likely to benefit from immunotherapy.

## Data Availability Statement

The original contributions presented in the study are included in the article/**Supplementary Material**. Further inquiries can be directed to the corresponding author.

## Ethics Statement

The studies involving human participants were reviewed and approved by The Ethical Committee of the School of Basic Medical Sciences, Shandong University. The patients/participants provided their written informed consent to participate in this study.

## Author Contributions

YF, JJ, and ZY designed the study. FL, LZ, WS, XC, and YW collected and analyzed the data and performed the expression verification of clinical samples. FL and YF wrote the manuscript. JJ and YF supervised the study. All authors contributed to the article and approved the submitted version.

## Funding

This study was supported by the National Natural Science Foundation of China (81971901 and 81772151), the Department of Science and Technology of Shandong Province (2018CXGC1208), the Natural Science Fund of Shandong Province (grant number ZR2020QH093), and the Shandong Provincial Key Laboratory of Immunohematology Open Research Program (grant number 2019XYKF006).

## Conflict of Interest

The authors declare that the research was conducted in the absence of any commercial or financial relationships that could be construed as potential conflicts of interest.

## Supplementary Material

Supplementary Figure 1 Identification of clinical feature-related modules through WGCNA and the expression heatmap of the 9 hub genes. **(A)** Dendrogram and clinical feature heatmap. The bottom panel shows the clinical characteristics of sex, age and stage. **(B, C)** Analysis of the average connectivity of the scale-free fitting index and various soft threshold functions. Assessment of the scale free topology when β = 9. **(D)** Hierarchical clustering dendrogram of similar genes based on topological overlapping. Genes with similar expression profiles are grouped into modules of the same color. **(E)** The heatmap shows the top 1000 genes of the topological overlap matrix (TOM) in the WGCNA, and the color degree is positively correlated with the degree of overlap. **(F)** The expression profiles of all genes in the midnight blue module.

Supplementary Figure 2 Analysis of the differential expression of 9 hub genes in gastric cancer tissues and normal tissues. **(A–I)** Differential expression box plots for *C1QA*, *C1QB*, *C1QC*, *CD14*, *CD163*, *CSF1R*, *FCER1G*, *MS4A6A* and *TYROBP*. All data were obtained from Gene Expression Profiling Interactive Analysis (GEPIA), *P < 0.05.

Supplementary Figure 3 The Kaplan-Meier survival curve showed the prognostic value of the 9 hub genes in gastric cancer. **(A–I)** Survival curves according to *C1QA*, *C1QB*, *C1QC*, *CD14*, *CD163*, *CSF1R*, *FCER1G*, *MS4A6A* and *TYROBP* expression. All data were from Kaplan-Meier Plotter.

Supplementary Figure 4 The identification of consensus clusters according to 9 hub genes and the consensus clustering results were evaluated, and the EMT markers and tumor purity were compared between the two clusters. **(A)** Consensus clustering matrix for k = 2. **(B)** The cumulative distribution function (CDF) of consensus clustering with k = 2 to 9. **(C)** Evaluation of the optimal number of clusters based on the NbClust package. **(D)** The classification results were further evaluated by the principal component analysis (PCA) method. **(E)** Analysis of the differential expression of several classic invasion and migration markers in two clusters of patients (cluster 1 n=132, cluster 2 n=116; Student’s t-tests). **(F)** Analysis of the difference in tumor purity scores between the two clusters (cluster 1 n=132, cluster 2 n=116; Student’s t-test). All data were from the GSE15460 dataset.

Supplementary Figure 5 Differential gene expression analysis and differential gene enrichment analysis of the two clusters. **(A)** Volcano map of differentially expressed genes between the two clusters (Student’s t-tests FDR < 0.05, log2fold change (FC) > 1). **(B)** Heatmap of differentially expressed genes between the two clusters (cluster 1 n=132, cluster 2 n=116). **(C)** Biological process (BP) enrichment analysis of differentially expressed genes. **(D)** Cellular component (CC) enrichment analysis of differentially expressed genes. **(E)** Molecular function (MF) enrichment analysis of differentially expressed genes. **(F)** KEGG enrichment analysis of differentially expressed genes between the two clusters. All data are from the GSE15460 dataset.

Supplementary Figure 6 Different datasets were applied to validate the multivariate Cox risk model. **(A, B)** Least absolute shrinkage and selection operator (LASSO) regression analysis. **(C)** Survival curves for different risk groups in the validation set (high-risk n=127, low-risk n=145; log-rank test). **(D)** Relationship between the patient survival time, survival status and risk score validation set (high-risk n=127, low-risk n=145). **(E)** Expression heatmap of 7 genes with different risk groups for the modeling validation set (high-risk n=127, low-risk n=145). **(F)** Time-dependent ROC analysis of the risk score in validation-set patients. **(G)** Overall survival curves in GSE84437 datasets based on the same cutoff value used to obtain the training set risk score (high-risk n=222, low-risk n=211; log-rank test). **(H)** Overall survival curves in TCGA datasets based on the same cutoff value used to obtain the training set risk score (high-risk n=136, low-risk n=187; log-rank test). **(I–J)** Time-dependent survival ROC curves for different datasets. Data for G are from the GSE84437 dataset, and those for H are from the TCGA dataset.

Supplementary Figure 7 Univariable Cox regression analyses of the risk scores and clinical factors in different validation datasets. **(A)** Internal validation dataset. **(B)** GSE15459. **(C)** GSE15460. **(D)** GSE84437. **(E)** Univariable Cox regression analyses based on overall survival (OS) in GSE62254. **(F)** Univariable Cox regression analyses based on disease-free survival (DFS) in GSE62254. **(G)** TCGA.

Supplementary Figure 8 Differential expression analysis of the 7 genes in gastric cancer tissues and normal tissues. **(A)** *APOD*. **(B)** *APOE*. **(C)** *CCDC80*. **(D)** *CTHRC1*. **(E)** *FERMT2*. **(F)** *GXYLT2*. **(G)** *SMPX*. **(H)** *APOD*. **(I)** *APOE*. **(J)** *CCDC80*. **(K)** *CTHRC1*. **(L)** *FERMT2*. **(M)** *GXYLT2*. **(N)** *SMPX*. A-G results from 16 pairs of cancer and adjacent tissues, and H-N data are from the GEPIA.

Supplementary Figure 9 The Kaplan-Meier survival curve shows the prognostic value of the 7 genes in gastric cancer. **(A)** *APOD*. **(B)** *APOE*. **(C)** *CCDC80*. **(D)** *CTHRC1*. **(E)** *FERMT2*. **(F)** *GXYLT2*. **(G)** *SMPX*. All data are from Kaplan-Meier Plotter.

Supplementary Figure 10 Copy number and mutation analysis of the 7 genes in The Cancer Genome Atlas (TCGA). **(A)** The circle graph shows the chromosomal locations of the 7 genes. **(B)** Genetic alteration of the 7 signature genes. The upper bar graph shows the survival status of each patient, and the lower bar graph shows the survival time of each patient.

Supplementary Figure 11 Gene mutation analysis was performed for high-risk and low-risk patients in TCGA. **(A)** The mutation map of the top 30 altered genes in the low-risk group. **(B)** The mutation map of the top 30 altered genes in the high-risk group. **(C)** Histogram of the difference in the tumor mutation burden (TMB) between the high- and low-risk groups (chi-square test).
