# Supplementary figures and images for: A Tumor Progression Related 7-Gene Signature Indicates Prognosis and Tumor Immune Characteristics of Gastric Cancer
Source: Front Oncol. 2021 Jun 14;11:690129. doi: 10.3389/fonc.2021.690129 (PMC8238374; doi:10.3389/fonc.2021.690129)

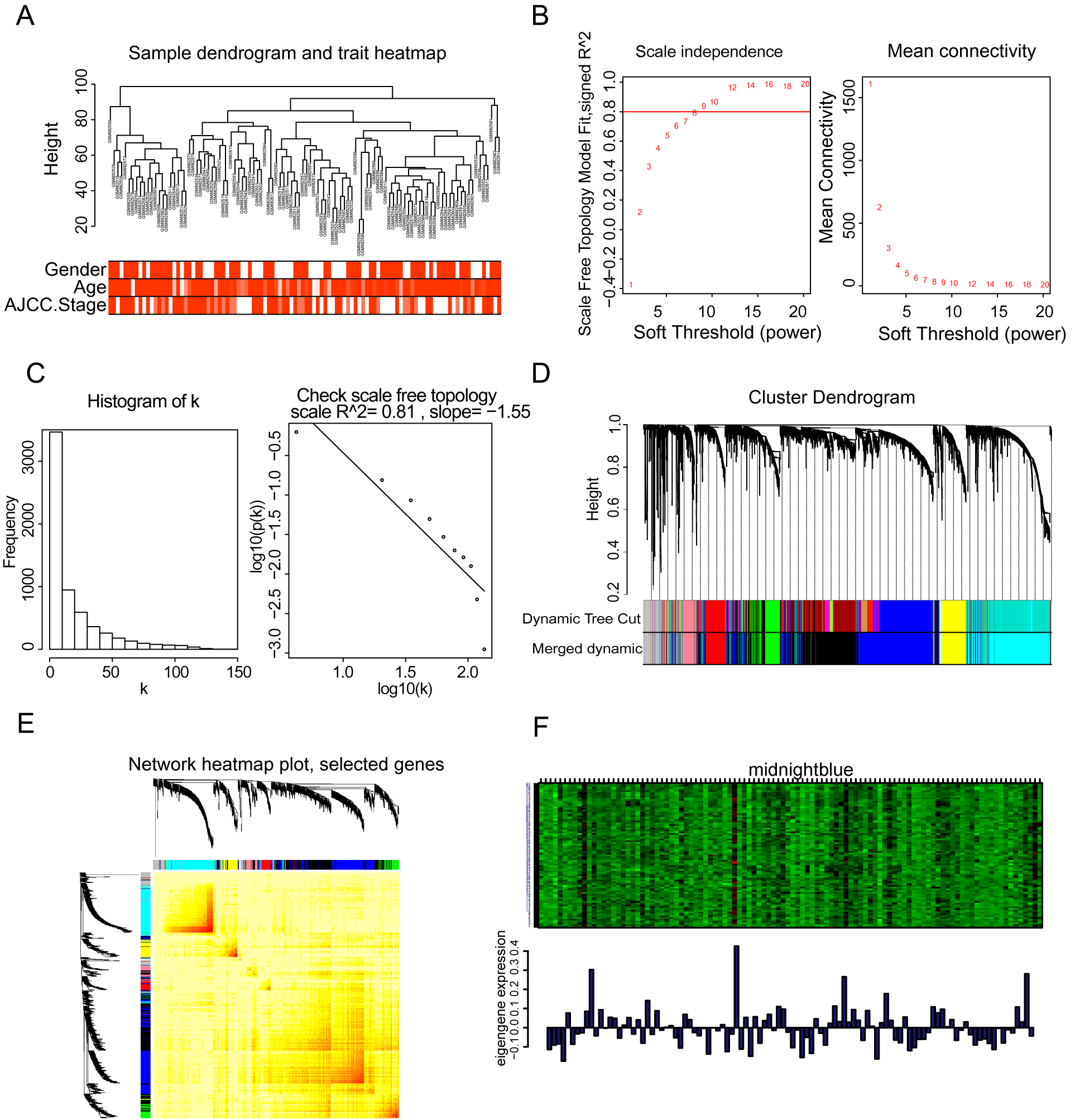

Supplement: Supplementary Figure 1 — Identification of clinical feature-related modules through WGCNA and the expression heatmap of the 9 hub genes. (A) Dendrogram and clinical feature heatmap. The bottom panel shows the clinical characteristics of sex, age and stage. (B, C) Analysis of the average connectivity of the scale-free fitting index and various soft threshold functions. Assessment of the scale free topology when β = 9. (D) Hierarchical clustering dendrogram of similar genes based on topological overlapping. Genes with similar expression profiles are grouped into modules of the same color. (E) The heatmap shows the top 1000 genes of the topological overlap matrix (TOM) in the WGCNA, and the color degree is positively correlated with the degree of overlap. (F) The expression profiles of all genes in the midnight blue module. [file Image_1.tif]

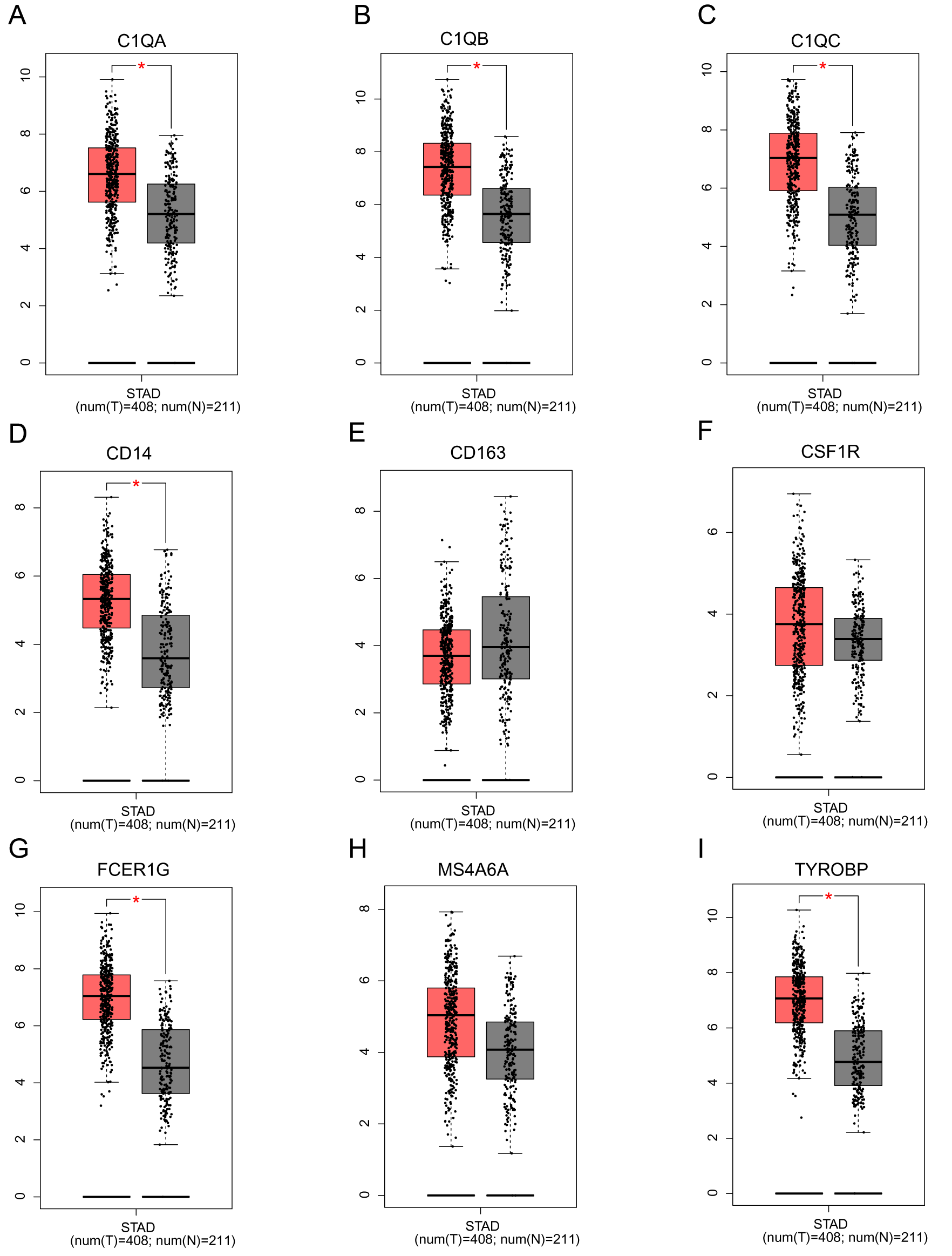

Supplement: Supplementary Figure 2 — Analysis of the differential expression of 9 hub genes in gastric cancer tissues and normal tissues. (A–I) Differential expression box plots for C1QA, C1QB, C1QC, CD14, CD163, CSF1R, FCER1G, MS4A6A and TYROBP. All data were obtained from Gene Expression Profiling Interactive Analysis (GEPIA), *P < 0.05. [file Image_2.tif]

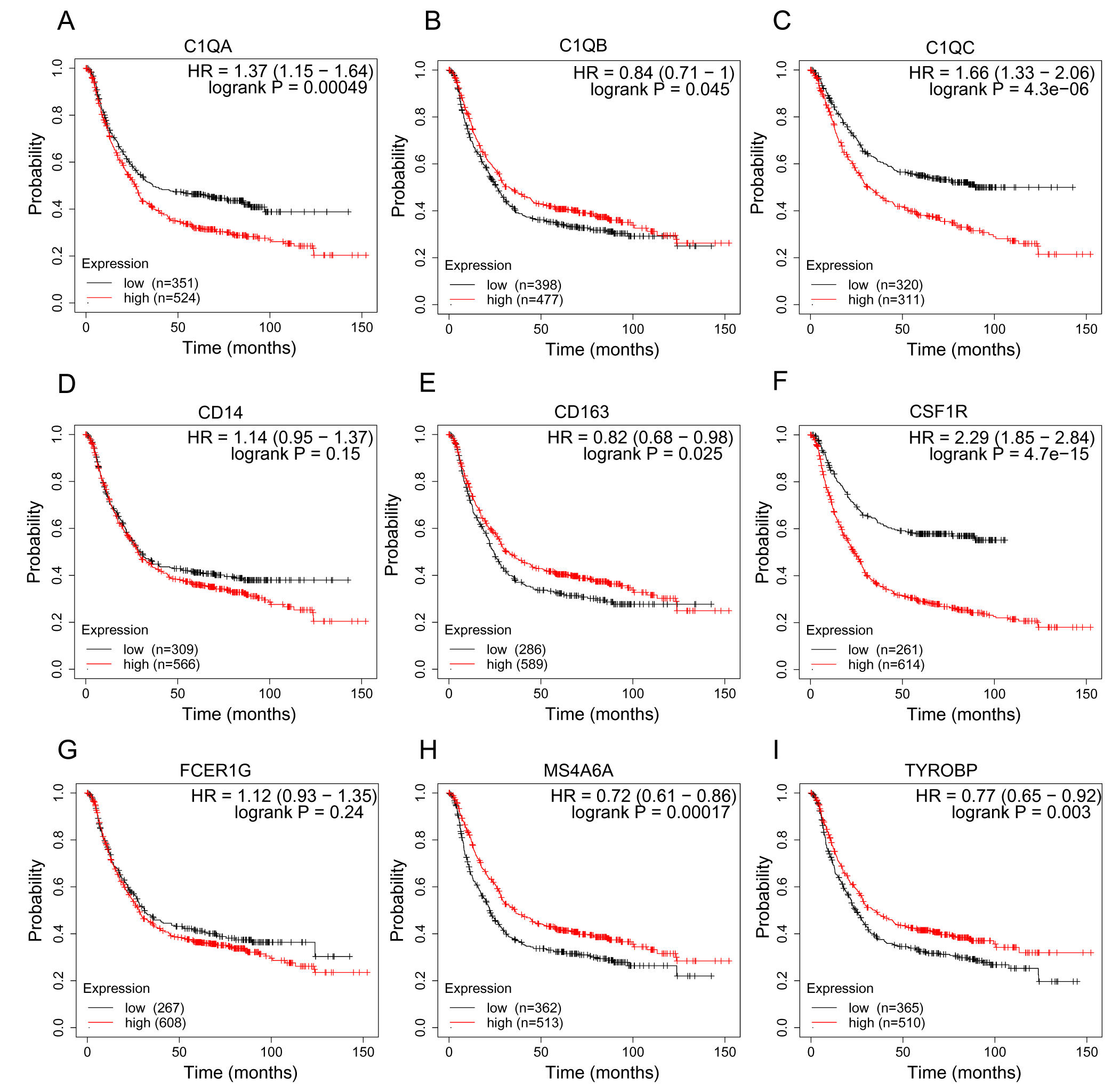

Supplement: Supplementary Figure 3 — The Kaplan-Meier survival curve showed the prognostic value of the 9 hub genes in gastric cancer. (A–I) Survival curves according to C1QA, C1QB, C1QC, CD14, CD163, CSF1R, FCER1G, MS4A6A and TYROBP expression. All data were from Kaplan-Meier Plotter. [file Image_3.tif]

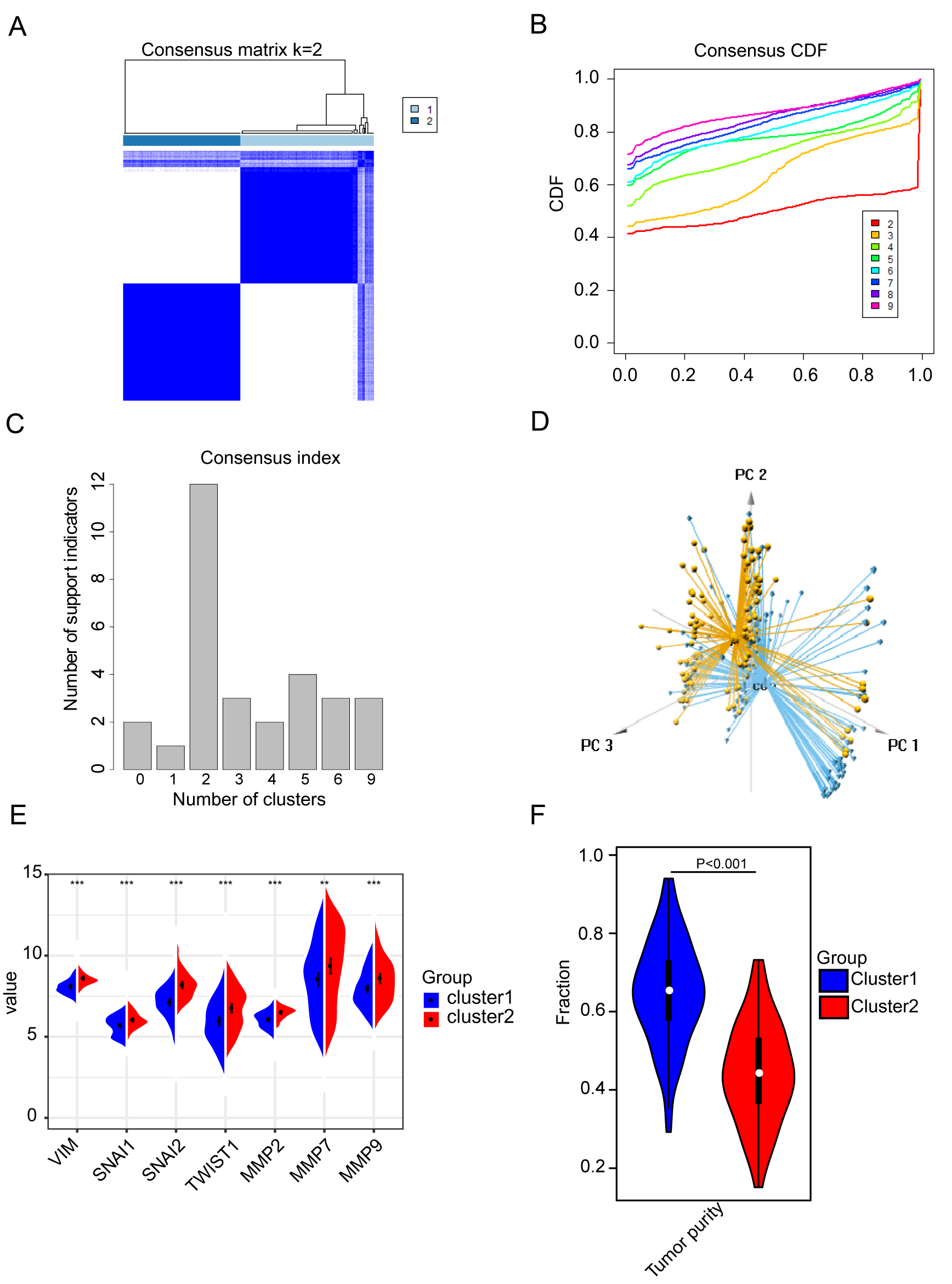

Supplement: Supplementary Figure 4 — The identification of consensus clusters according to 9 hub genes and the consensus clustering results were evaluated, and the EMT markers and tumor purity were compared between the two clusters. (A) Consensus clustering matrix for k = 2. (B) The cumulative distribution function (CDF) of consensus clustering with k = 2 to 9. (C) Evaluation of the optimal number of clusters based on the NbClust package. (D) The classification results were further evaluated by the principal component analysis (PCA) method. (E) Analysis of the differential expression of several classic invasion and migration markers in two clusters of patients (cluster 1 n=132, cluster 2 n=116; Student’s t-tests). (F) Analysis of the difference in tumor purity scores between the two clusters (cluster 1 n=132, cluster 2 n=116; Student’s t-test). All data were from the GSE15460 dataset. [file Image_4.tif]

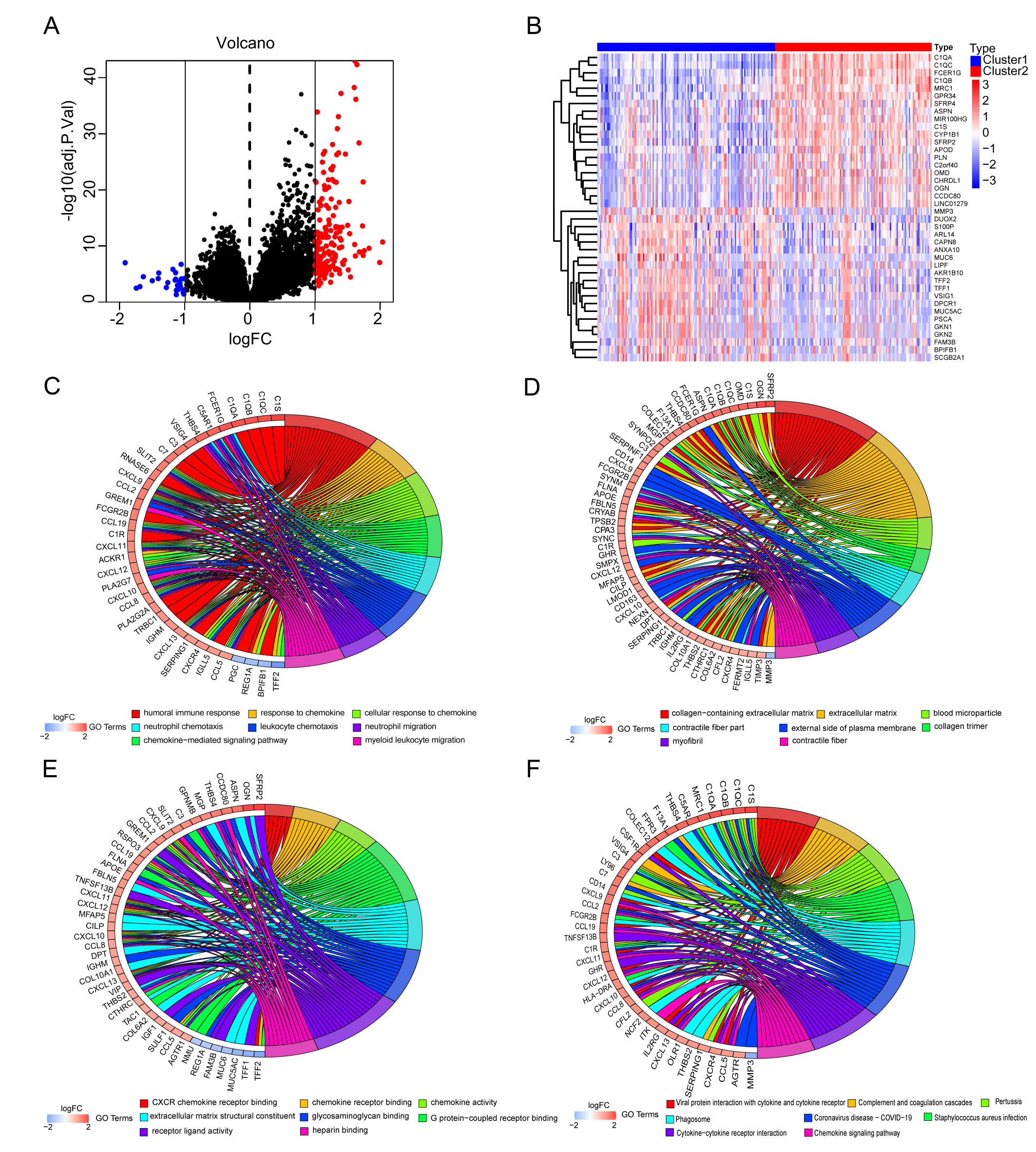

Supplement: Supplementary Figure 5 — Differential gene expression analysis and differential gene enrichment analysis of the two clusters. (A) Volcano map of differentially expressed genes between the two clusters (Student’s t-tests FDR < 0.05, log2fold change (FC) > 1). (B) Heatmap of differentially expressed genes between the two clusters (cluster 1 n=132, cluster 2 n=116). (C) Biological process (BP) enrichment analysis of differentially expressed genes. (D) Cellular component (CC) enrichment analysis of differentially expressed genes. (E) Molecular function (MF) enrichment analysis of differentially expressed genes. (F) KEGG enrichment analysis of differentially expressed genes between the two clusters. All data are from the GSE15460 dataset. [file Image_5.tif]

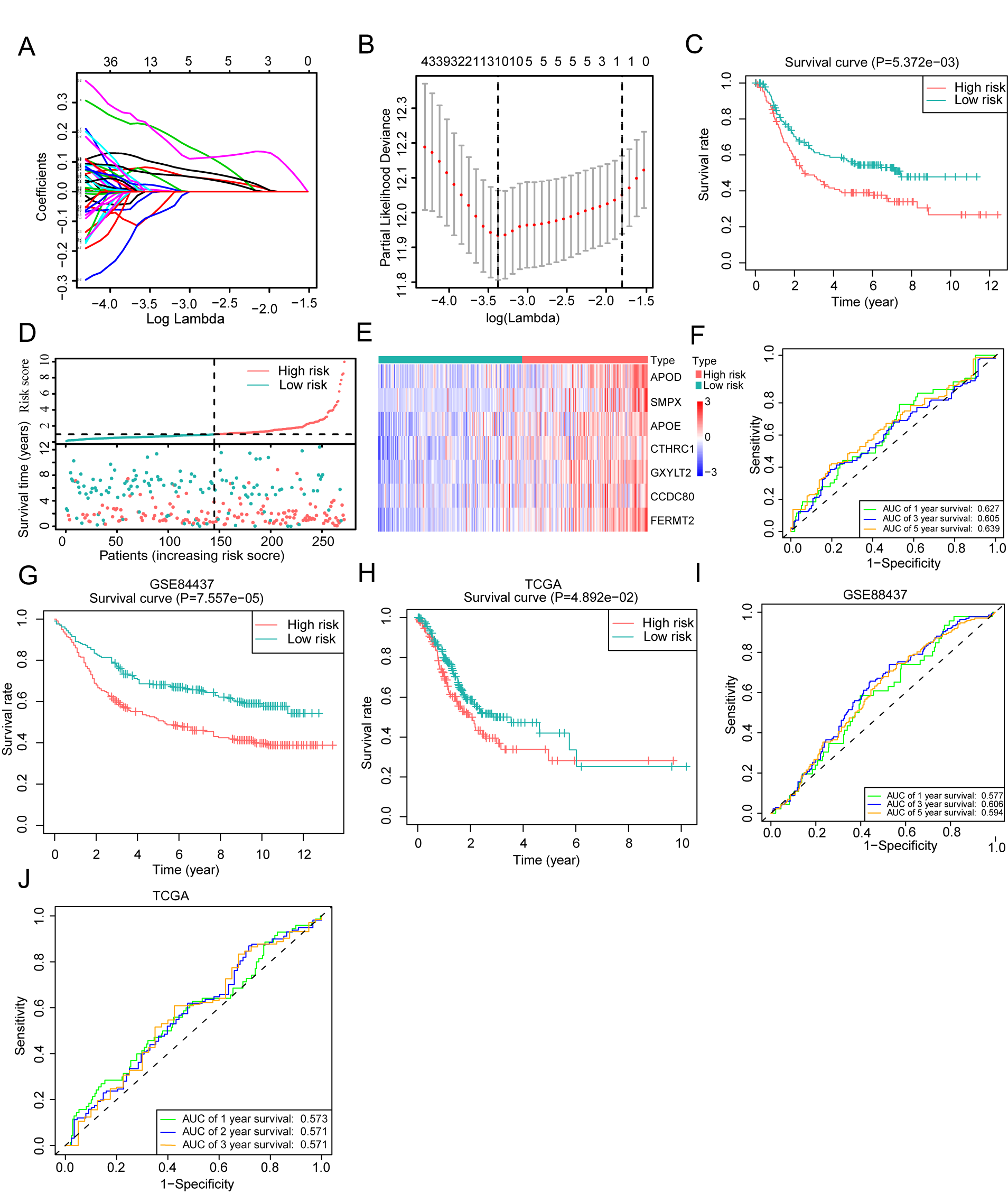

Supplement: Supplementary Figure 6 — Different datasets were applied to validate the multivariate Cox risk model. (A, B) Least absolute shrinkage and selection operator (LASSO) regression analysis. (C) Survival curves for different risk groups in the validation set (high-risk n=127, low-risk n=145; log-rank test). (D) Relationship between the patient survival time, survival status and risk score validation set (high-risk n=127, low-risk n=145). (E) Expression heatmap of 7 genes with different risk groups for the modeling validation set (high-risk n=127, low-risk n=145). (F) Time-dependent ROC analysis of the risk score in validation-set patients. (G) Overall survival curves in GSE84437 datasets based on the same cutoff value used to obtain the training set risk score (high-risk n=222, low-risk n=211; log-rank test). (H) Overall survival curves in TCGA datasets based on the same cutoff value used to obtain the training set risk score (high-risk n=136, low-risk n=187; log-rank test). (I–J) Time-dependent survival ROC curves for different datasets. Data for G are from the GSE84437 dataset, and those for H are from the TCGA dataset. [file Image_6.tif]

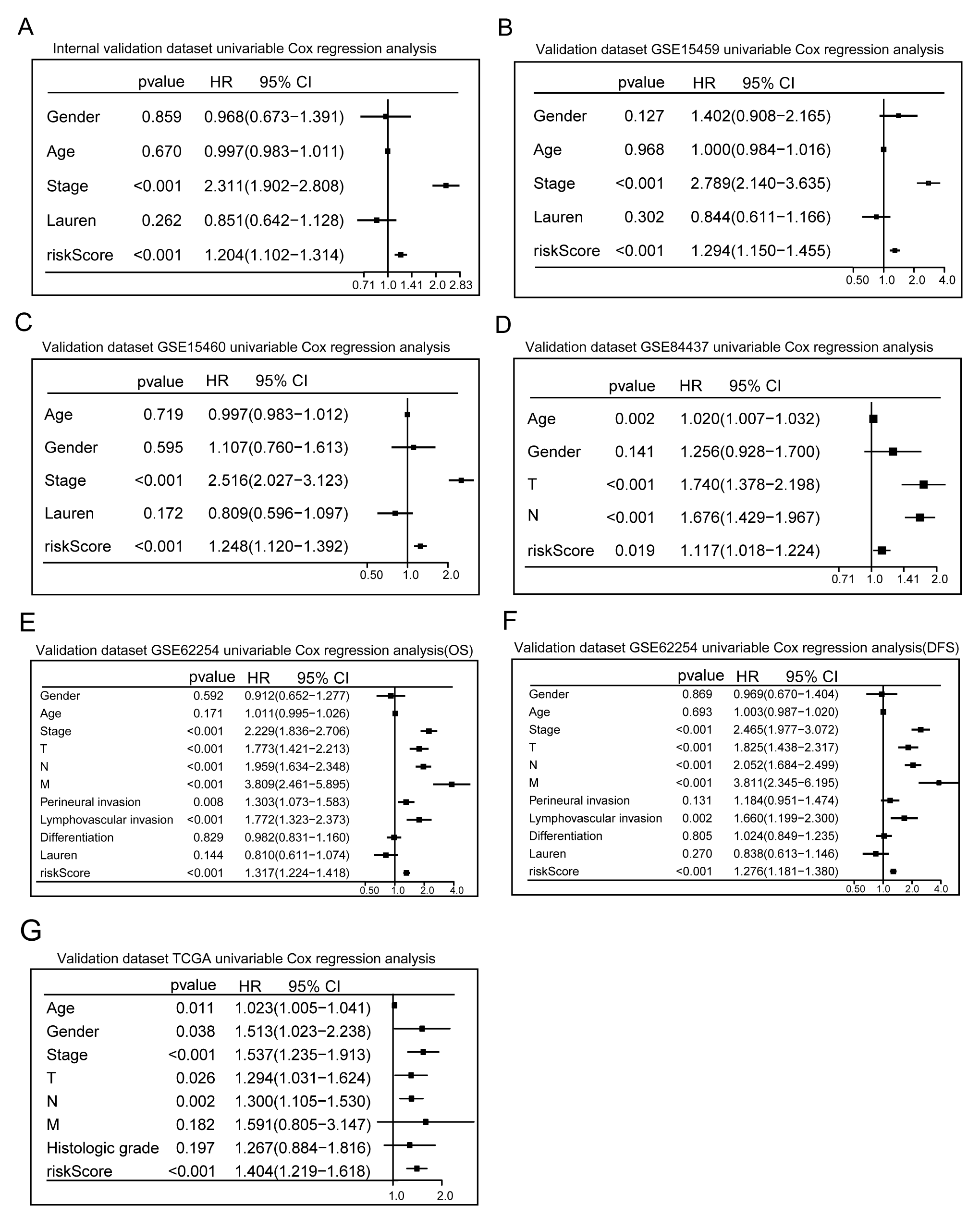

Supplement: Supplementary Figure 7 — Univariable Cox regression analyses of the risk scores and clinical factors in different validation datasets. (A) Internal validation dataset. (B) GSE15459. (C) GSE15460. (D) GSE84437. (E) Univariable Cox regression analyses based on overall survival (OS) in GSE62254. (F) Univariable Cox regression analyses based on disease-free survival (DFS) in GSE62254. (G) TCGA. [file Image_7.tif]

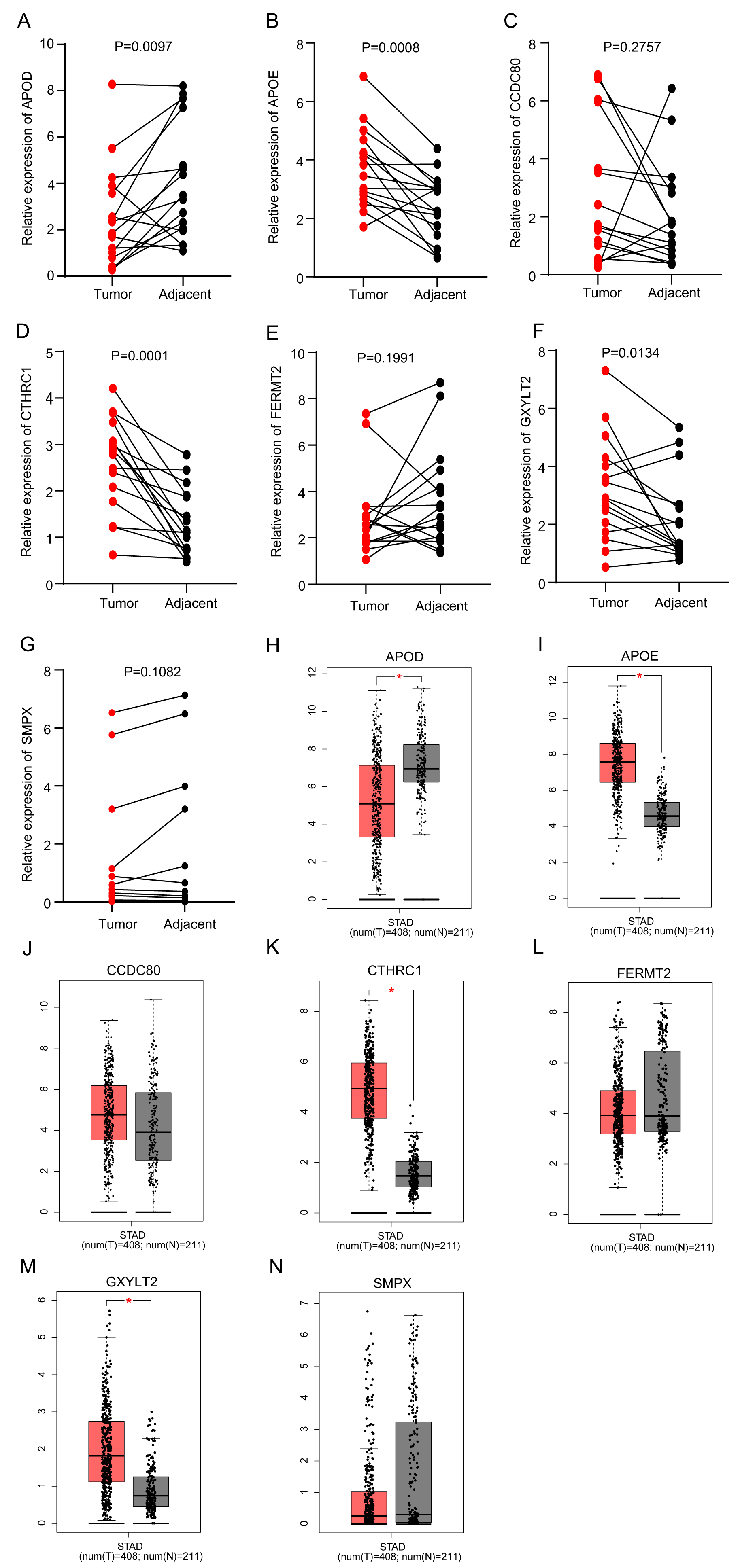

Supplement: Supplementary Figure 8 — Differential expression analysis of the 7 genes in gastric cancer tissues and normal tissues. (A) APOD. (B) APOE. (C) CCDC80. (D) CTHRC1. (E) FERMT2. (F) GXYLT2. (G) SMPX. (H) APOD. (I) APOE. (J) CCDC80. (K) CTHRC1. (L) FERMT2. (M) GXYLT2. (N) SMPX. A-G results from 16 pairs of cancer and adjacent tissues, and H-N data are from the GEPIA. [file Image_8.tif]

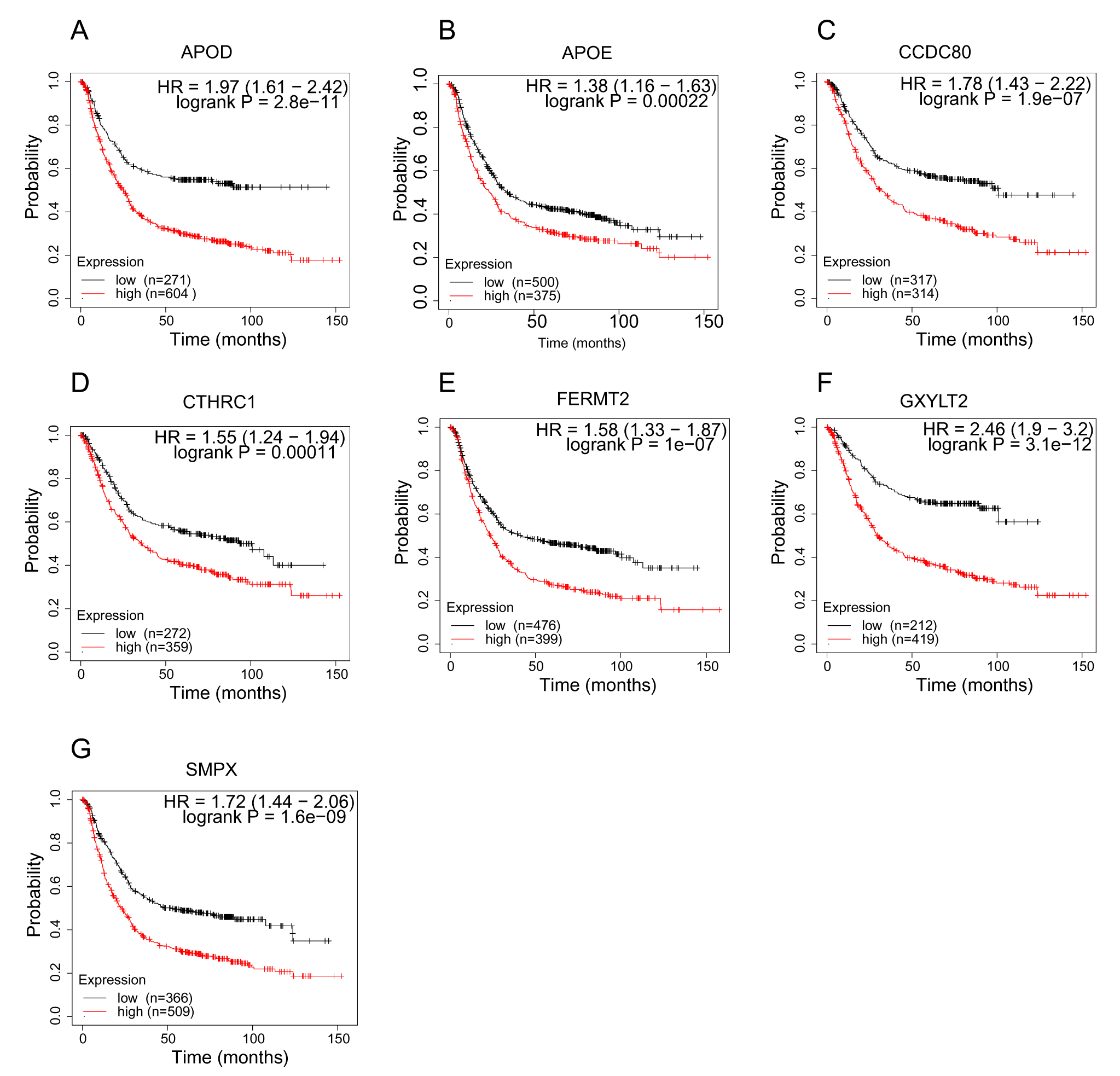

Supplement: Supplementary Figure 9 — The Kaplan-Meier survival curve shows the prognostic value of the 7 genes in gastric cancer. (A) APOD. (B) APOE. (C) CCDC80. (D) CTHRC1. (E) FERMT2. (F) GXYLT2. (G) SMPX. All data are from Kaplan-Meier Plotter. [file Image_9.tif]

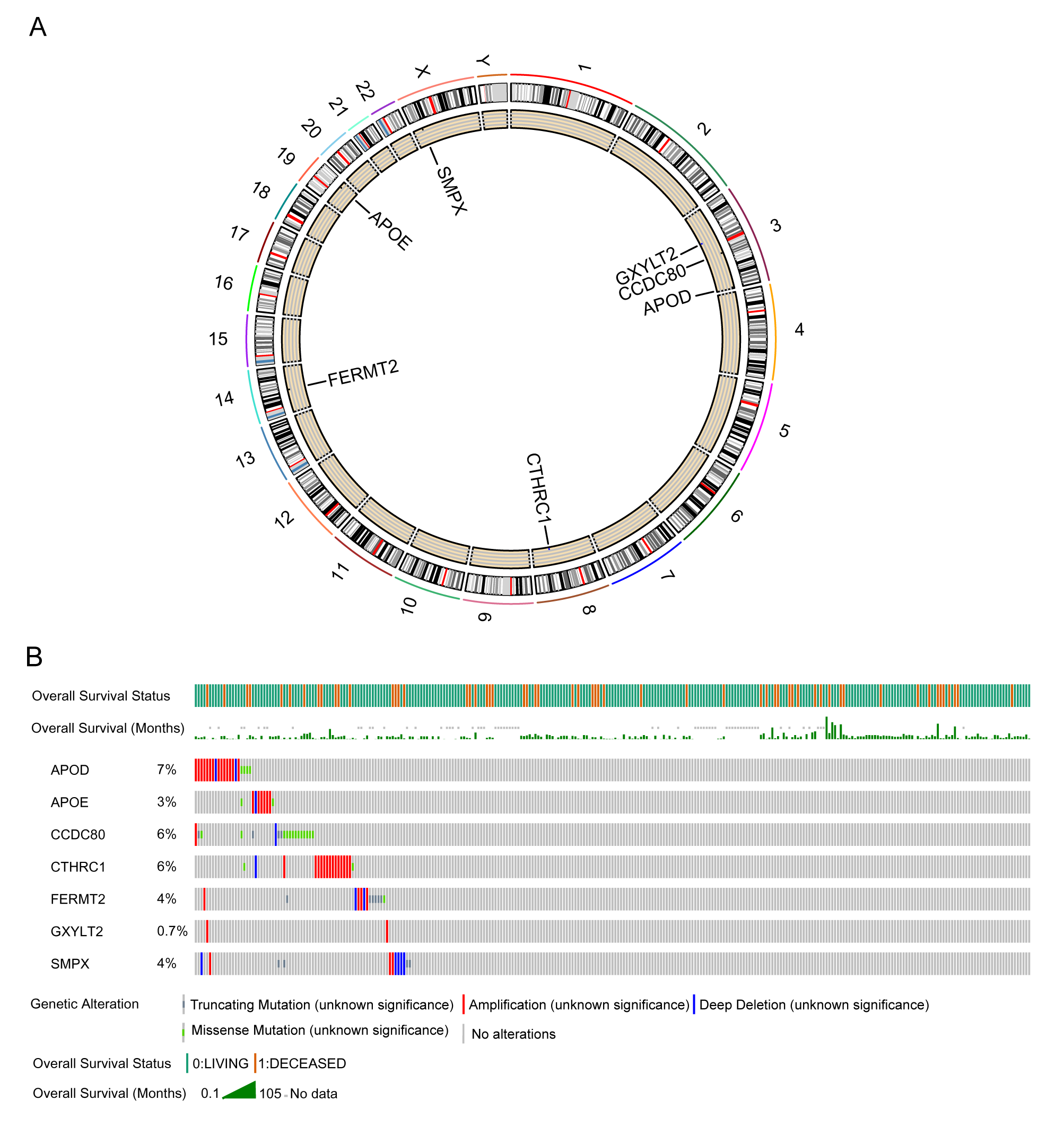

Supplement: Supplementary Figure 10 — Copy number and mutation analysis of the 7 genes in The Cancer Genome Atlas (TCGA). (A) The circle graph shows the chromosomal locations of the 7 genes. (B) Genetic alteration of the 7 signature genes. The upper bar graph shows the survival status of each patient, and the lower bar graph shows the survival time of each patient. [file Image_10.tif]

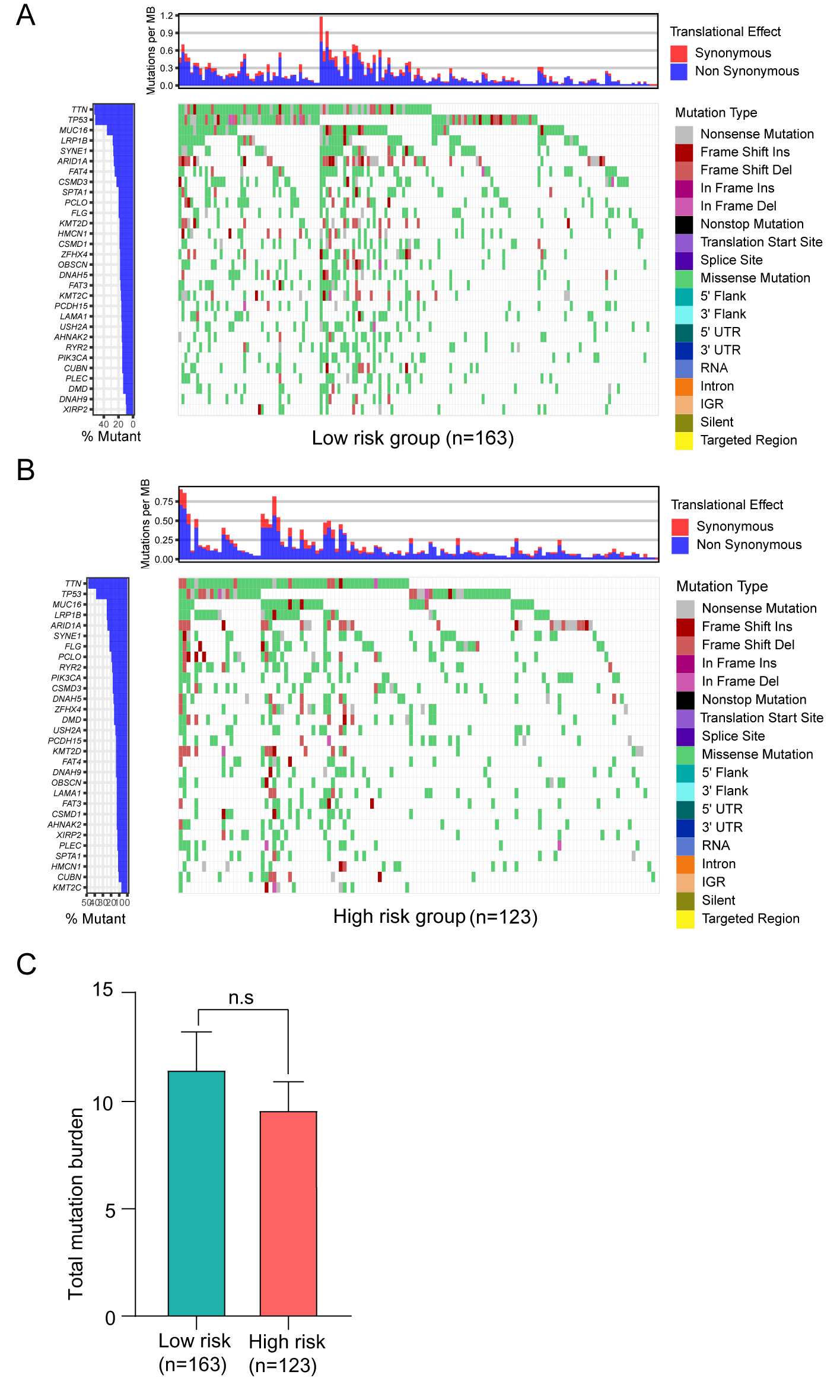

Supplement: Supplementary Figure 11 — Gene mutation analysis was performed for high-risk and low-risk patients in TCGA. (A) The mutation map of the top 30 altered genes in the low-risk group. (B) The mutation map of the top 30 altered genes in the high-risk group. (C) Histogram of the difference in the tumor mutation burden (TMB) between the high- and low-risk groups (chi-square test). [file Image_11.tif]
